# AARS1‐mediated AKR1B10 lactylation stabilizes an aerobic glycolysis‐positive feedback loop to drive lenvatinib resistance in hepatocellular carcinoma

**DOI:** 10.1002/ctm2.70561

**Published:** 2025-12-26

**Authors:** Zijian Liu, Jingsheng Yuan, Shitong Su, Jiaqi Han, Ni Zeng, Yuhan Ma, Nianyong Chen, Tao Lv

**Affiliations:** ^1^ Laboratory of Cancer Therapy and Immunity Department of Radiation Oncology and Department of Head and Neck Oncology Cancer Center West China Hospital Sichuan University Chengdu China; ^2^ Department of Liver Transplantation Center & Institute of Organ Transplantation West China Hospital Sichuan University Chengdu China; ^3^ Key Laboratory of Transplant Engineering and Immunology NHC West China Hospital Sichuan University Chengdu China; ^4^ Department of Pathology West China Second University Hospital Sichuan University Chengdu China

**Keywords:** aerobic glycolysis, epalrestat, lactylation, lenvatinib resistance, ubiquitination

## Abstract

**Background:**

Lenvatinib resistance (LR) represents a significant obstacle in hepatocellular carcinoma (HCC) treatment. Aldo‐keto reductase family 1 member B10 (AKR1B10) is involved in tumour metabolic reprogramming; however, its role in LR remains unclear.

**Methods:**

Bioinformatics analyses of public databases were integrated and validated in established LR HCC cell lines. Functional assays (CCK‐8, flow cytometry and Seahorse XF analysis) were performed to assess proliferation, apoptosis and aerobic glycolysis. Post‐translational modifications of AKR1B10 were characterized using co‐immunoprecipitation, mass spectrometry and western blot.

**Results:**

AKR1B10 was identified as a critical driver of resistance by establishing a metabolic positive feedback loop. Bioinformatics analyses and experimental validation demonstrated that AKR1B10 upregulation correlates with therapeutic resistance. Functional studies indicated that AKR1B10 promotes resistance by enhancing aerobic glycolysis. Mechanistically, alanyl‐tRNA synthetase 1 mediates lactylation modification at AKR1B10 lysine 173 (K173), stabilizing AKR1B10 by blocking ubiquitin (Ub)‐proteasomal degradation. Stabilized AKR1B10 interacts physically with lactate dehydrogenase A (LDHA), promoting LDHA phosphorylation at Y10 and accelerating glycolytic lactate production. The increased lactate subsequently induces histone H3K18 lactylation (H3K18la), which transcriptionally upregulates LDHA expression. Thus, a self‐reinforcing AKR1B10–lactate–LDHA amplification circuit is formed. Clinical analyses confirmed elevated AKR1B10 expression in LR HCC patient tissues. Importantly, targeting this axis with the AKR1B10 inhibitor epalrestat (EPA) synergized with lenvatinib, overcoming resistance in xenograft mouse models and patient‐derived xenograft models.

**Conclusions:**

These findings establish AKR1B10 as both a biomarker and a therapeutic target in HCC. They reveal a novel lactylation‐driven glycolytic adaptation mechanism and support the clinical translation of combined EPA–lenvatinib therapy.

**Key points:**

- AKR1B10 confers lenvatinib resistance by enhancing aerobic glycolysis in HCC cells.
- AKR1B10 undergoes AARS1‐mediated lactylation at K173, stabilizing it by antagonizing ubiquitin‐proteasomal degradation.
- AKR1B10 promotes LDHA Y10 phosphorylation, boosting lactate production, which drives H3K18la‐mediated transcriptional upregulation of LDHA, creating a feed‐forward loop.
- Targeting AKR1B10 with epalrestat synergizes with lenvatinib to overcome resistance in preclinical models.

## Graphical abstract

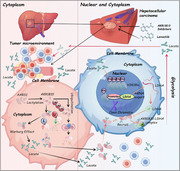

## 1 INTRODUCTION

As the most prevalent subtype of primary liver cancer, hepatocellular carcinoma (HCC) is a significant global health challenge. It is responsible for more than 80% of cases and is epidemiologically ranked as the sixth most common cancer diagnosis and the third greatest contributor to cancer‐associated mortality.^1^, ^2^ Surgical resection or liver transplantation can potentially cure patients, yet many individuals are diagnosed at advanced disease stages, making these surgical approaches unsuitable and substantially limiting available therapeutic strategies.^3^ Lenvatinib, a frontline treatment for advanced‐stage HCC, functions as a multi‐targeted tyrosine kinase inhibitor (TKI) against various targets such as vascular endothelial growth factor receptor 1‐3 (VEGFR1‐3), fibroblast growth factor receptor 1‐4 (FGFR1‐4), platelet‐derived growth factor receptor alpha (PDGFRα), KIT proto‐oncogene receptor tyrosine kinase (KIT) and RET proto‐oncogene receptor tyrosine kinase (RET).^4^, ^5^ However, its effectiveness is limited by low response rates^6^ and the development of intrinsic or acquired resistance, which presents a significant clinical obstacle. Therefore, clarifying the molecular mechanisms underlying lenvatinib resistance (LR) is critical for improving therapeutic strategies and patient outcomes.

In addition to its roles in cellular detoxification and lipogenesis through the stabilization of ACCα,^7^, ^8^, ^9^ Aldo‐keto reductase family 1 member B10 (AKR1B10) is markedly overexpressed in HCC tissues, including early‐stage and Alpha‐fetoprotein (AFP)‐negative cases, which supports its utility as an effective serum biomarker for diagnosis.^10^ AKR1B10 overexpression has been documented in various malignancies, including liver,^11^, ^12^, ^13^, ^14^ lung,^15^ breast^16^ and pancreatic cancers,^17^ where it enhances tumour growth and metastasis.^18^, ^19^, ^20^ Specifically, AKR1B10 was reported as overexpressed in lung cancer brain metastasis, associated with pemetrexed resistance,^21^ and also mediated resistance to radiotherapy via the free fatty acid (FFA)/toll‐like receptor 4 (TLR4)/nuclear factor kappa‐B (NF‐κB) axis in nasopharyngeal carcinoma.^22^ Importantly, AKR1B10 facilitates chemoresistance through multiple mechanisms, including the detoxification of carbonyl‐containing drugs, amplification of the Warburg effect (via ACCα stabilization and glycolytic gene upregulation) and enhancement of redox homeostasis. Nevertheless, the complex molecular mechanisms regulating AKR1B10 during LR remain unclear, although metabolic reprogramming may be involved.

Recent studies highlight metabolic reprogramming—especially enhanced glycolysis (Warburg effect)—as critical for resistance to TKIs in HCC.^23^ Intriguingly, post‐translational modifications (PTMs), particularly lactylation—a novel epigenetic modification derived from lactate—have emerged as regulators of oncogenic signalling and drug resistance.^24^, ^25^, ^26^ Elevated lactate in the tumour microenvironment induces histone and non‐histone protein lactylation, altering protein function and stability. Preliminary evidence indicates that AKR1B10 may undergo lactylation, potentially enhancing its enzymatic activity or stability. This modification could establish a feed‐forward loop wherein AKR1B10‐driven glycolysis elevates lactate production, subsequently lactylating and potentiating AKR1B10 to further amplify glycolysis. This hyperactivated glycolytic state likely fuels energy production, maintains redox balance and supports biosynthetic pathways, enabling HCC cells to evade lenvatinib‐induced cytotoxicity. Therefore, investigating how AKR1B10 lactylation promotes glycolysis‐mediated LR in HCC is crucial.

Central to this mechanism is the amplification of glycolytic flux that produces excessive lactate. Lactate‐driven protein lactylation serves as a critical molecular bridge. Lactate sensors and lactyltransferases, such as alanyl‐tRNA synthetase 1 (AARS1), transfer lactate groups and catalyse adenosine triphosphate (ATP)‐dependent lysine lactylation on proteins. These mechanisms converge into a unified model wherein lactate overproduction orchestrates resistance through metabolic adaptation and epigenetic reprogramming.^27^ Lactate dehydrogenase A (LDHA) plays a pivotal role in this axis. Proteomic analyses of lenvatinib‐resistant models indicate LDHA co‐enrichment with glycolytic enzymes, where LDHA converts pyruvate to lactate, fuelling histone and non‐histone lactylation.^28^, ^29^ Notably, phosphorylation at LDHA Y10 enhances its enzymatic activity,^30^ yet the upstream kinase regulating this phosphorylation site in HCC resistance remains unidentified.

This study identifies AKR1B10 as a metabolic linchpin driving LR through a self‐amplifying lactate‐glycolysis circuit. Integrated bioinformatics analyses and functional validation reveal that AKR1B10 potentiates glycolytic flux, establishing a feed‐forward loop. Mechanistically, AARS1‐mediated lactylation at AKR1B10 lysine 173 (K173) stabilizes AKR1B10 by preventing Ub‐dependent degradation. Stabilized AKR1B10 reciprocally interacts with LDHA, promoting its phosphorylation at Y10 and accelerating lactate production. Increased lactate then drives histone H3K18 lactylation (H3K18la), transcriptionally upregulating LDHA and maintaining sustained glycolytic hyperactivity. Therapeutically, targeting this axis with epalrestat (EPA) disrupts the resistance circuit, synergizing with lenvatinib to restore drug sensitivity in vivo. These results reveal AARS1‐dependent lactylation as a targetable metabolic vulnerability and support co‐inhibition of AKR1B10 as a promising strategy to overcome LR in HCC.

## 2 MATERIALS AND METHODS

### 2.1 Public data processing

To establish a comprehensive dataset, we first acquired clinical, transcriptomic and copy number variation data from The Cancer Genome Atlas (TCGA). This was complemented by proteomic and clinical datasets extracted from published supplementary materials.^31^ From the Gene Expression Omnibus, we downloaded single‐cell RNA sequencing (GSE166635),^32^ H3K18la chromatin immunoprecipitation (ChIP)‐seq (GSE156674),^33^ and RNA‐seq data comparing lenvatinib‐resistant to parental Huh7 cells (GSE211850).^34^ Additionally, gene expression patterns of AKR family members in HCC versus normal tissue were analysed using combined TCGA and Genotype Tissue Expression (GTEx) data accessed through Sanger Box. Baseline AKR1B10 expression across multiple datasets and spatial transcriptomics data were also obtained from the HCCDB online repository.

### 2.2 Clinical samples and immunohistochemistry (IHC)

Freshly collected HCC specimens (*n* = 161) from patients at West China Hospital, Sichuan University, from May 2014 to December 2020, underwent retrospective analysis. Among these, 40 patients who had received lenvatinib treatment were categorized into two groups, including 15 partial response (PR) and 25 progressive disease (PD), based on the modified Response Evaluation Criteria in Solid Tumours (mRECIST 1.1). The IHC staining was scored independently by two observers, considering both the intensity and distribution of staining. The staining levels were defined based on the proportion of tumour cells showing positive staining (negative: < 5%; low: 5%–25%; medium: 26–75%; high: > 75%). Based on this assessment, samples were categorized as having either high or low AKR1B10 expression. Specifically, those with medium or high staining intensity were grouped as high expression, while those with negative or low intensity were deemed low expression. Subsequent survival analysis of the HCC cohort was performed using this dichotomized classification.

### 2.3 Cell culture and reagents

The human HCC cell lines Hep3B (RRID: CVCL_0326), Huh7 (RRID: CVCL_0336), murine Hepa1‐6 (RRID: CVCL_0327) and human embryonic kidney 293T (RRID: CVCL_0063) were sourced from the Cell Bank of the Chinese Academy of Sciences. These cells were maintained in dulbecco's modified eagle's medium (DMEM) (Gibco) supplemented with 10% fetal bovine serum (FBS) and 1% penicillin‐streptomycin (HyClone) at 37°C in a 5% CO_2_ atmosphere. All cell lines were routinely authenticated via short tandem repeat (STR) profiling and tested to be free of mycoplasma contamination. Key reagents, including lenvatinib, MG132, cycloheximide (CHX), puromycin and EPA, were procured from Selleckchem, while rotenone and 2‐deoxy‐D‐glucose (2‐DG) were obtained from Beyotime.

### 2.4 Lentivirus construction and transfection

HEK293T cells underwent co‐transfection with lentiviral vectors and packaging plasmids psPAX2 and pMD2G. Stable cell lines were selected after 2 weeks of puromycin treatment. Lentiviral vectors (pLV3‐puro) utilized in the study were procured from MiaoLingBio. Full‐length open reading frames of AKR1B10, LDHA and AARS1 were amplified via RT‐PCR (polymerase chain reaction) and subsequently cloned into expression vectors. These recombinant vectors were transfected into HCC cells with Lipofectamine 3000 (Thermo Fisher), following the manufacturer's recommended protocol. Additionally, Ub sequences were cloned into the GV141‐His vector. Site‐specific and deletion mutants were created using the QuikChange site‐directed mutagenesis kit (Stratagene). Transfection methods were previously described,^35^, ^36^ with transfection efficiency confirmed through RT‐qPCR and western blotting (WB) analyses conducted 24 h post transfection. The sequences of shRNAs targeting AKR1B10, AARS1 and LDHA were as follows:

Sh‐AKR1B10‐1: 5′‐ GTGCCTATGTCTATCAGAA ‐3′;
Sh‐AKR1B10‐2: 5′‐ CACGCATTGTTGAGA ACAT ‐3′;
Sh‐AARS1‐1: 5′‐ GGTGGATGACAGCAGTGAAGA ‐3′;
Sh‐AARS1‐2: 5′‐ GGACATCATTAATGAAGAAGA ‐3′;
Sh‐LDHA‐1: 5′‐ GGATATACCAACTGGGCTA ‐3′;
Sh‐LDHA‐2: 5′‐ GTACAGTCCTGATTGCATC ‐3′;
Negative Control (NC): 5′‐ TTCTCCGAACGTGTCACGT ‐3′.

### 2.5 Real‐time quantitative PCR (qRT‐PCR)

Total RNA was isolated using TRIzol reagent (Invitrogen), and cDNA was synthesized from the extracted RNA with a High‐Capacity cDNA Reverse Transcription Kit (Takara) according to the manufacturer's instructions. Quantitative PCR was then performed using a TBGreen kit (Takara) to measure mRNA levels, which were calculated via the 2^−ΔΔCT^ method with normalization to glyceraldehyde‐3‐phosphate dehydrogenase (GAPDH). The primer sequences used are listed below:

AKR1B10 forward: 5′‐ CCCAGGAGACAGAGGTTATA ‐3′;
AKR1B10 reverse: 5′‐ GAAATGATTCTGAGTGAGCAGGTAG ‐3′;
LDHA forward: 5′‐ TCGGCTCAAGCAGATGGG ‐3′;
LDHA reverse: 5′‐ AGTGGGCGTCAGTGGGTGT ‐3′;
GAPDH forward: 5′‐ACCCAGAAGACTGTGGATGG‐3′;
GAPDH reverse: 5′‐CAGTGAGCTTCCCGTTCAG‐3′.

### 2.6 ChIP

ChIP assays were conducted following the manufacturer's protocol (Guangzhou Saicheng Biotechnology Co. Ltd). The supernatants were immunoprecipitated with an anti‐H3K18la antibody (PTM Bio, PTM‐1406) at 4°C overnight. The purified DNA fragments were then analysed by quantitative real‐time PCR (qPCR) using the following primers:

AKR1B10‐site‐1 forward: 5′‐ ACATTGGTGCTGGGATTACA ‐3′;
AKR1B10‐site‐1 reverse: 5′‐ CCAGTGCAGCTTTGAGATAGA ‐3′;
AKR1B10‐site‐2 forward: 5′‐ TGGCATACTCAAGGGCTTAC ‐3′;
AKR1B10‐site‐2 reverse: 5′‐ TACCTCTGGGCCTGTATTCT ‐3′;
AKR1B10‐site‐3 forward: 5′‐ CCACTGACAGTTCTTGGGTATG ‐3′;
AKR1B10‐site‐3 reverse: 5′‐ CAGGGTGACTCAGTTCAAATGTA ‐3′;
AKR1B10‐site‐4 forward: 5′‐ GGAGTAGCTCAGAGTGATCTTG ‐3′;
AKR1B10‐site‐4 reverse: 5′‐ TCGACGTTTGGGTGTAAGTATAG ‐3′;
AKR1B10‐site‐5 forward: 5′‐ CATGGCAGCTTTGACCTACT ‐3′;
AKR1B10‐site‐5 reverse: 5′‐ ATATTGGTGCACGCCTGTAG ‐3′;
AKR1B10‐site‐6 forward: 5′‐ CACCTGAGGTCAGTAGTTCAAG ‐3′;
AKR1B10‐site‐6 reverse: 5′‐ CACCACACCTGGCTAATTCT ‐3′.

### 2.7 Western blot analysis

Total proteins were extracted from cultured cells with radioimmunoprecipitation assay buffer (RIPA) lysis buffer (Millipore) supplemented with proteinase and phosphatase inhibitors (both from Sigma‐Aldrich). The extracted proteins were then subjected to western blot analysis using the specified primary antibodies, with ACTB (β‐actin) serving as the loading control. Protein signals were visualized using an enhanced chemiluminescence detection kit (Meilunbio) and quantified by densitometry with ImageJ software. The primary antibodies used in this study were as follows: anti‐AKR1B10, anti‐AARS1, anti‐LDHA, anti‐p‐LDHA (Y10) (Affinity, America), anti‐HA, anti‐Flag, anti‐His (HUABIO), anti‐Kla, anti‐H3K18la (PTM BIO) and anti‐β‐actin (BOSTER).

### 2.8 Cell viability assay

Cells were seeded in 96‐well plates at 1000 cells per well and allowed to adhere overnight. Following treatment with the specified doses of lenvatinib, cell viability was assessed on designated days by adding 10 µL of Cell Counting Kit‐8 (CCK‐8) solution (Biosharp Biotechnology) to each well. After a 2‐h incubation at 37°C, the absorbance at 450 nm was measured using a spectrophotometer.

### 2.9 Flow cytometry analysis

Flow cytometry analysis was performed to detect the cell cycle distribution and apoptosis rate of each sample using Apoptosis Kit (Elabscience). The cells were collected 24 h after treated with lenvatinib, washed with PBS and resuspended in 200 µL of Annexin V Binding Buffer. After 2.5 µL of Annexin V‐FITC Reagent and 2.5 µL of PI Reagent were added, the cells were incubated at room temperature in the dark for 15–20 min. Cell staining was detected using a CytoFLEX flow cytometer, and the apoptosis rate was analysed with FlowJo 10.4.0 software.

### 2.10 Measurement of ATP levels, glucose uptake and lactate production

Intracellular metabolic states were assessed using commercial kits according to manufacturers' protocols. ATP levels were quantified using an ATP Assay Kit (Beyotime, China), with luminescence measured in opaque‐walled 96‐well plates. Glucose concentration was assayed with a corresponding kit (Beyotime, China) by measuring the absorbance at 630 nm after adding the reaction reagent, and calculated against a standard curve. Similarly, lactic acid production was determined using a specific kit (Solarbio), with the absorbance of the supernatant at 570 nm being proportional to its concentration, which was also quantified via a standard curve.

### 2.11 Seahorse assay

The indicated cells were plated in an XF96 plate (Seahorse Biosciences) measured with a Seahorse XFe96 Analyzer (Agilent) to analyse the extracellular acidification rate (ECAR). After basal readings were obtained, rotenone/antimycin A (Rot/AA, 0.5 mM) and 2‐DG (50 mM) were injected in sequence. All drugs were provided in the Seahorse XF Glycolytic Rate Assay Kit (Agilent, 103344‐100).

### 2.12 CoIP and MS analysis

CoIP assays were performed using a CoIP kit (Abs955, Absin) according to the manufacturer's recommended protocol, as described before.^37^ The mass spectrometry (MS) analysis was conducted by Bioprofile. The ubiquitin (Ub) assay was conducted under denaturing conditions as previously described.^38^ The Fast Silver Stain Kit (Beyotime) was used to conduct silver staining and the proteins of interest obtained using MS were further detected by WB.

### 2.13 Animal studies

All animal experiments were conducted in accordance with the NIH Guide for the Care and Use of Laboratory Animals and were approved by the Animal Care and Use Committee of West China Hospital, Sichuan University (Approval No. 2020351A). Six‐week‐old female BALB/c nude and C57BL/6 mice, sourced from HFK Bioscience, were maintained under specific pathogen‐free conditions with a 12‐h light/dark cycle and ad libitum access to food and water. For subcutaneous xenograft models, 5 million pre‐treated Huh7 or Hepa1‐6 cells were injected into BALB/c nude or C57BL/6 mice, respectively. Treatment with EPA and lenvatinib was initiated on Day 5 post injection and administered by oral gavage every other day for 2 weeks. Patient‐derived xenograft (PDX) models were established by subcutaneously implanting 2–3 mm^3^ fragments of fresh human HCC tissue into non‐obese diabetic / severe combined immunodeficiency (NOD/SCID) mice. Upon reaching 1–2 cm^3^, the tumours were harvested and injected into BALB/c nude mice. When tumour volumes reached approximately 100 mm^3^, mice received EPA and lenvatinib via gavage every other day for 3 weeks. Tumour dimensions were measured every other day with callipers, and volumes were calculated using the formula: Volume = 1/2 × (longest diameter) × (shortest diameter)^2^. Mice were euthanized at designated time points, and excised tumours were weighed, fixed in 4% formaldehyde and paraffin‐embedded for subsequent analysis.

### 2.14 Enzyme activity and supernatant concentration assays of AKR1B10

Supernatants from the designated cell cultures were harvested for the measurement of AKR1B10 concentration using enzyme‐linked immunosorbent assay (ELISA) kits (USCN). Following the kit protocol, samples were first centrifuged and incubated, after which specific reaction reagents and solution buffers were added sequentially. Absorbance at 450 nm was recorded with a microplate reader (Tecan). The reductase activity in the specified cells was evaluated by monitoring the decline in absorbance of nicotinamide adenine dinucleotide phosphate (NADPH) at 340 nm. The assay system contained phosphate‐buffered saline (pH 7.0), 20 mM DL‐glyceraldehyde,.3 mM NADPH and 50 µg of soluble protein—normalized according to the AKR1B10 levels in the supernatants. The reduction in optical density at 340 nm was continuously measured over 20 min at 37°C using the same microplate reader. One unit (U) of enzyme activity was defined as the quantity of enzyme required to oxidize 1 µM of NADPH per minute under 37°C.

### 2.15 Generation of AKR1B10^−/−^ cell lines

AKR1B10 knockout cell lines were generated using the CRISPR‐Cas9 system. The single‐guide RNAs (sgRNAs), designed using the CRISPOR tool (https://crispor.gi.ucsc.edu/), included the targeting sequence (GATAAAGGTAATGCCATCGG) and a non‐targeting control (TTCTTAGAAGTTGCTCCAC). The selection was based on high predicted on‐target efficiency and minimal off‐target potential scores provided by the tool. These oligonucleotides were cloned into the pSpCas9(BB)‐2A‐Puro (PX459) vector (Addgene #48139). Subsequently, Huh7 and Hep3B cells were transfected with the resultant plasmids using Lipofectamine 3000 (Thermo Fisher Scientific). For lentiviral delivery, HEK293T cells were co‐transfected with the sgRNA vectors and packaging plasmids (psPAX2, Addgene #12260; pMD2.G, Addgene #12259). The viral supernatant was harvested 72 h post‐transfection, and transduced cells were selected with puromycin. Successful knockout was confirmed by WB.

### 2.16 Statistical analysis

Protein docking between AKR1B10 and its interactors (AARS1, LDHA) was initially predicted using the AlphaFold Web Server and further refined with Discovery Studio software. Statistical analyses were performed as follows: Spearman's correlation for coefficient calculation; the Kaplan–Meier method with log‐rank testing for survival analysis; receiver operating characteristic (ROC) curves to evaluate the diagnostic power of AKR genes; Student's *t*‐test and one‐way ANOVA for normally distributed data; Wilcoxon and Kruskal–Wallis tests for non‐parametric data; and the chi‐square test for clinical correlations. The mean fluorescence intensity from immunofluorescence was quantified using ImageJ. Information on AKR1B10 PTMs was retrieved from the publicly accessible PhosphoSitePlus database (https://www.phosphosite.org/homeAction). Data are presented as mean ± SD from at least three independent experiments. A two‐sided *p*‐value < .05 defined statistical significance.

## 3 RESULTS

### 3.1 Aerobic glycolysis contributes to LR in HCC

**FIGURE 1:**
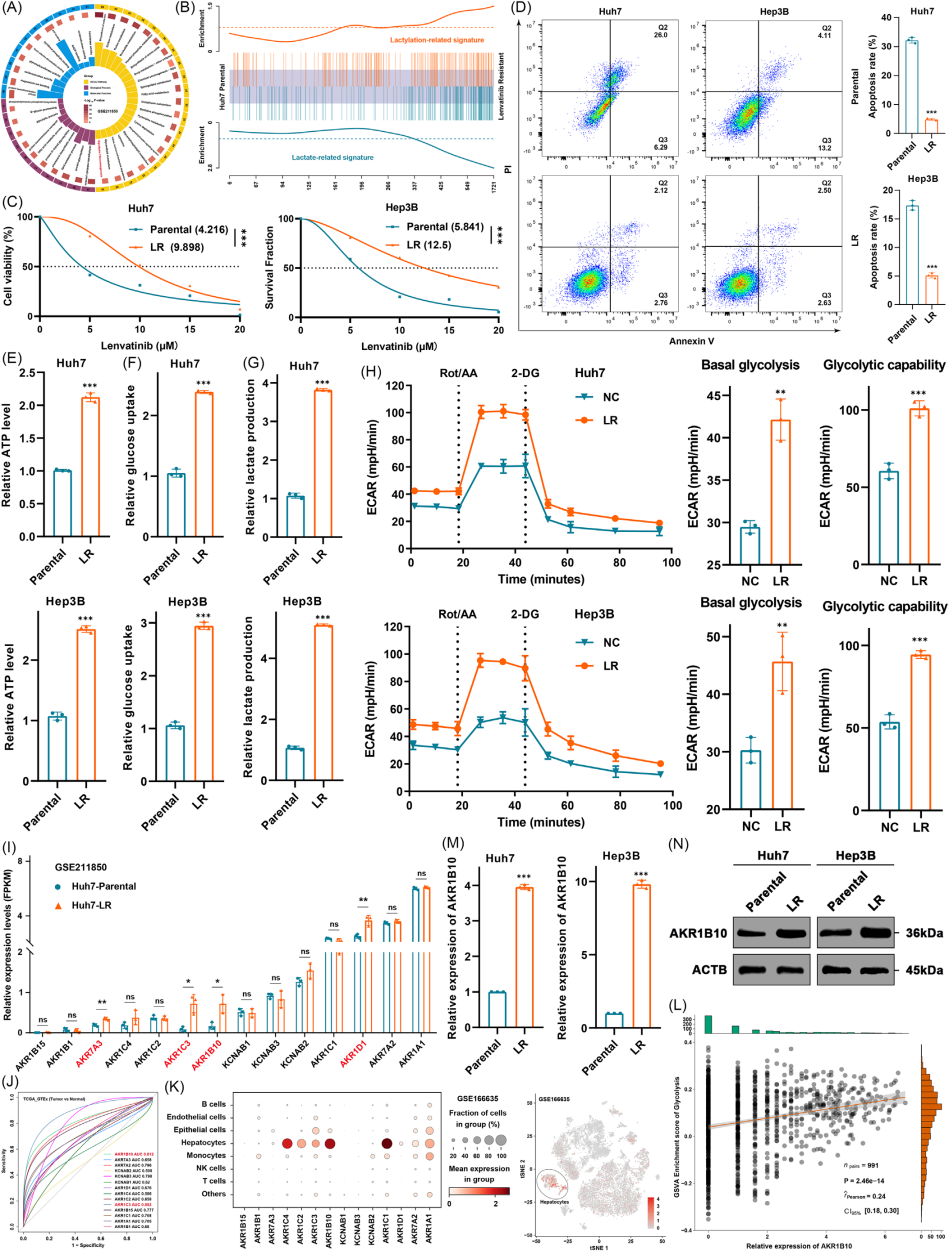
Aerobic glycolysis contributes to lenvatinib resistance (LR) in hepatocellular carcinoma (HCC). (A) Gene ontology enrichment analysis of differentially expressed genes between parental and LR Huh7 cells (GSE211850). (B) Gene set enrichment analysis for lactylation‐related and lactate‐related gene signatures in parental versus LR Huh7 cells (GSE211850). (C) IC50 values of lenvatinib in parental and LR Huh7 and Hep3B cell lines after 72‐h treatment. (D) Apoptosis rates in parental and LR Huh7 and Hep3B cells following lenvatinib treatment. (E–G) Intracellular ATP levels, intracellular glucose levels and extracellular lactate production in parental versus LR HCC cells. (H) Seahorse extracellular acidification rate (ECAR) profiles comparing basal glycolysis and glycolytic capacity in parental versus LR HCC cells. (I) Relative mRNA expression levels of aldo‐keto reductases (AKRs) in parental versus LR Huh7 cells (GSE211850). (J) Receiver operating characteristic curve analysis evaluating the diagnostic potential of AKRs for HCC using The Cancer Genome Atlas (TCGA) and Genotype Tissue Expression datasets. (K) Expression distribution of AKRs across cell populations in scRNA‐seq dataset GSE166635. Right panel: Distribution of aldo‐keto reductase family 1 member B10 (AKR1B10) expression among HCC sub‐populations. (L) Correlation analysis between AKR1B10 expression and glycolysis gene set enrichment scores (GSE166635). (M, N) qRT‐PCR and WB analysis of basal AKR1B10 expression levels in parental versus LR HCC cells. Data are mean ± SD; **p < .01, ***p < .001.

To investigate the mechanisms underlying LR in HCC, we analysed the public dataset GSE211850. Differential gene expression and gene ontology enrichment analyses were performed between parental and LR Huh7 cells (Figure 1A). These analyses revealed the glycolysis and gluconeogenesis signalling pathways as a crucial kyoto encyclopedia of genes and genomes (KEGG) pathway enriched in LR phenotypes. Consistent with this, gene set enrichment analysis indicated an association between LR and lactate generation in HCC (Figure 1B). To validate this hypothesis, we established LR HCC cell lines (Huh7 and Hep3B). Half maximal inhibitory concentration (IC50) assays confirmed significantly higher resistance in LR cells, compared to parental cells (Figure 1C). Furthermore, lenvatinib treatment induced significantly less apoptosis in LR cells than in parental cells (Figure 1D), confirming the successful generation of LR models. Aerobic glycolysis, characterized by increased glucose uptake and lactate production, has been linked to resistance against targeted therapies.^39^ To assess its role in LR, we measured cellular ATP levels, glucose uptake and extracellular lactate levels in parental and LR cells. As expected, LR cells exhibited higher ATP levels, increased glucose consumption and elevated lactate production (Figure 1E–G). Seahorse analysis further revealed enhanced ECARs, including basal glycolysis and glycolytic capacity (Figure 1H), suggesting that increased glycolysis is a common feature of LR.

Aldo‐keto reductases (AKRs) are cytosolic enzymes catalyzing cofactor‐dependent redox reactions.^40^ To explore their potential role in LR, we re‐analysed GSE211850 data to compare AKRs expression between parental and LR Huh7 cells (Figure 1I). This showed significantly higher mRNA expression of AKR7A3, AKR1C3, AKR1B10 and AKR1D1 in LR cells. To systematically evaluate AKRs across cancers, particularly HCC, we performed multi‐omics bioinformatics analyses using public data. Integrating transcriptional data from GTEx and TCGA, we profiled the expression of fourteen AKRs across 25 tumour types (Figure S1A). AKRs were specifically elevated in most digestive system neoplasms, especially HCC and cholangiocarcinoma (CHOL), and generally higher in tumours than normal tissues, suggesting AKRs may function as initiating oncogenes promoting tumour progression. To assess the diagnostic potential of AKRs in HCC, ROC analysis showed that only AKR1B10 and AKR1C3 had area under the curve values ≥.8 (Figure 1J), indicating good diagnostic value. Univariate Cox analysis identified only AKR1B10 and AKR1C3 as statistically significant risk factors for poorer overall survival (OS), progression‐free survival (PFS) and disease‐free survival (DFS) in HCC patients (Figure S1B). Analysis of public proteomic data confirmed significantly higher protein levels of AKR1B10 and AKR1C3 in HCC tissues, compared to adjacent normal tissues (Figure S1C). To determine tissue specificity, we re‐analysed scRNA‐seq data (GSE166635). AKR1B10 expression in hepatocytes was higher than AKR1C3, and most AKRs were predominantly expressed in hepatocytes (Figure 1K). Correlation analysis revealed a significant positive association between AKR1B10 expression and glycolysis gene set enrichment scores (Figure 1L), suggesting its involvement in HCC glycolysis. Analysis of 14 HCC datasets within the HCCDB database consistently showed significantly higher AKR1B10 mRNA expression in HCC than in adjacent tissues (Figure S2A). Spatial transcriptomics data (HCCDB) also confirmed uniformly higher AKR1B10 expression in tumour regions versus para‐tumour regions (Figure S2B–D). Critically, qRT‐PCR and WB verified significantly higher mRNA and protein levels of AKR1B10 in LR cells, compared to parental cells (Figure 1M,N). Therefore, we identified AKR1B10 as a candidate gene for further investigation into its functional role in LR.

### 3.2 AKR1B10 mediates LR depending on aerobic glycolysis

**FIGURE 2:**
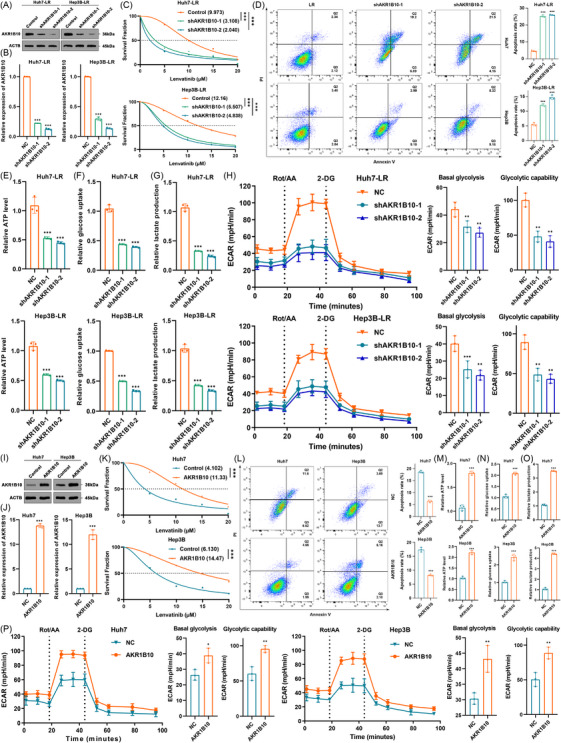
AKR1B10 mediates LR depending on aerobic glycolysis. (A‐B) qRT‐PCR and WB assessing AKR1B10 knockdown efficiency in HCC cells. (C) IC50 values of lenvatinib in LR HCC cells with stable AKR1B10 knockdown. (D) Apoptosis rates in AKR1B10‐knockdown LR HCC cells after lenvatinib treatment. (E–G) Intracellular ATP levels, intracellular glucose levels and extracellular lactate production in AKR1B10‐knockdown LR HCC cells. (H) ECAR profiles comparing basal glycolysis and glycolytic capacity in AKR1B10‐knockdown LR HCC cells. (I‐J) qRT‐PCR and WB validating AKR1B10 overexpression efficiency in HCC cells. (K) IC50 values of lenvatinib in HCC cells with stable AKR1B10 overexpression. (L) Apoptosis rates in AKR1B10‐overexpressing HCC cells treated with lenvatinib. (M–O) Intracellular ATP levels, intracellular glucose levels and extracellular lactate production in AKR1B10‐overexpressing HCC cells. (P) ECAR profiles comparing basal glycolysis and glycolytic capacity in AKR1B10‐overexpressing HCC cells. Data are mean ± SD; *p < .05, **p < .01, ***p < .001.

To confirm the stimulatory role of AKR1B10 in LR progression in HCC, we stably knocked down AKR1B10 in Huh7 and Hep3B LR cell lines. qRT‐PCR and WB confirmed efficient knockdown (Figure 2A,B). As expected, AKR1B10 suppression significantly enhanced lenvatinib sensitivity, evidenced by reduced IC50 values and increased apoptosis in LR cells (Figure 2C,D). Consistent with this, AKR1B10 knockdown in LR cells decreased cellular ATP levels, glucose uptake and extracellular lactate levels, indicating inhibition of aerobic glycolysis (Figure 2E–G). Seahorse analysis further revealed decreased ECAR, including basal glycolysis and glycolytic capacity, upon AKR1B10 knockdown (Figure 2H). Conversely, we stably overexpressed AKR1B10 in parental HCC cells, with qRT‐PCR and WB verifying overexpression efficiency (Figure 2I,J). Overexpression of AKR1B10 significantly increased IC50 values and reduced lenvatinib‐induced apoptosis (Figure 2K,L). Notably, stable AKR1B10 expression enhanced aerobic glycolysis in HCC cells as shown by increased cellular ATP levels, glucose uptake, extracellular lactate levels and glycolytic capacity (Figure 2M–P). Collectively, these findings demonstrate that AKR1B10 promotes LR and regulates aerobic glycolysis in HCC.

To further elucidate whether aerobic glycolysis is essential for AKR1B10‐mediated LR, we treated cells with the glycolysis inhibitor 2‐DG. Adding 2‐DG partially reversed the enhanced LR conferred by AKR1B10 overexpression, decreasing IC50 values and increasing apoptosis (Figure S3A,B). Moreover, 2‐DG counteracted the stimulatory effect of AKR1B10 overexpression on aerobic glycolysis, reducing ATP levels, glucose uptake and lactate production (Figure S3C–E). Conversely, the glycolysis activator rotenone increased IC50 values and decreased apoptosis in AKR1B10‐knockdown cells (Figure S3F,G). Furthermore, rotenone partially rescued the inhibitory effects of AKR1B10 knockdown on aerobic glycolysis (Figure S3H–J). Together, these results indicate that AKR1B10 promotes LR, at least partially, through the regulation of aerobic glycolysis.

### 3.3 Lactylation at Lys173 enhances AKR1B10 protein expression

**FIGURE 3:**
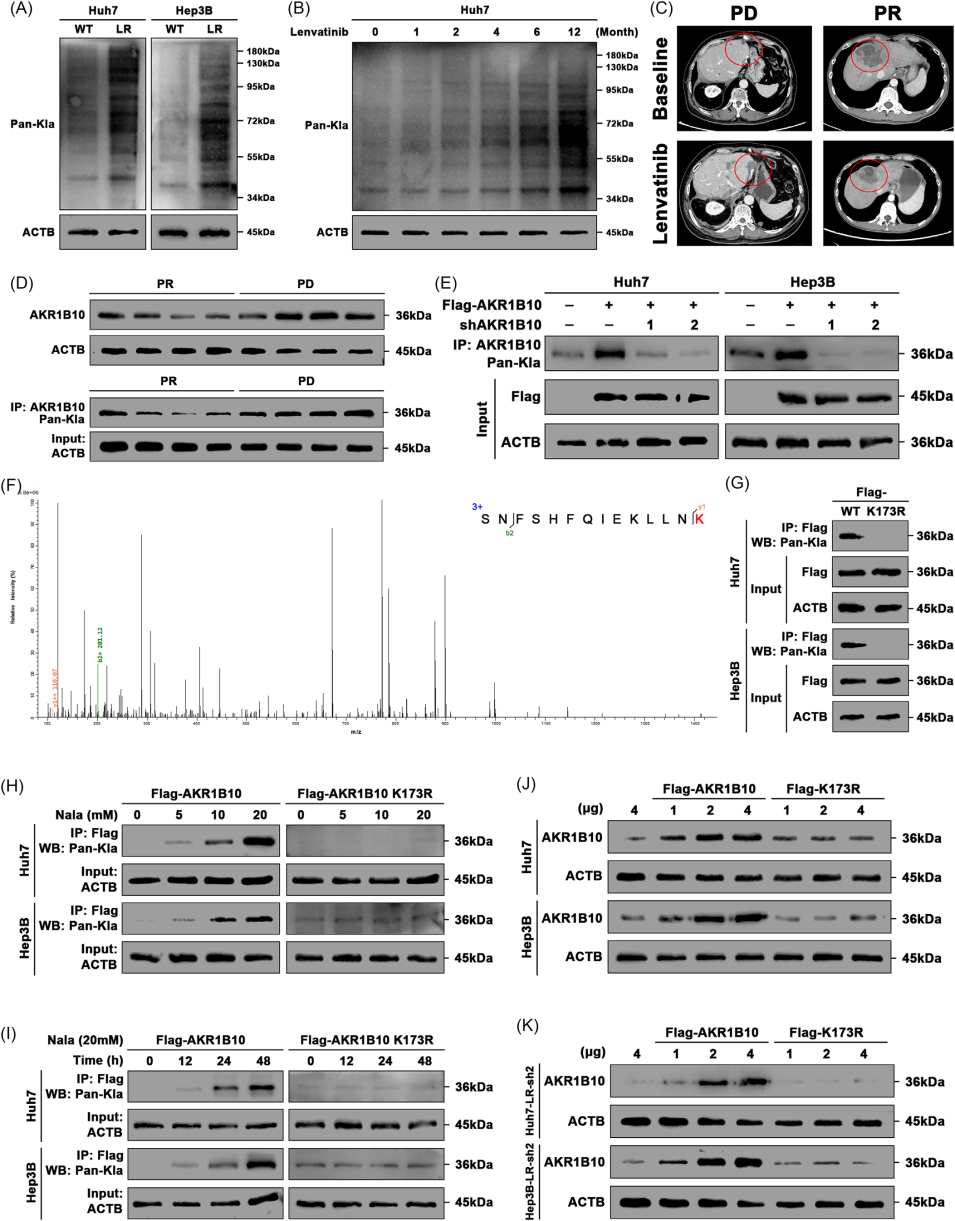
Lactylation at Lys173 enhances AKR1B10 protein expression. (A) Western blot analysis of pan‐lysine lactylation (pan‐Kla) levels in parental versus LR HCC cells. (B) Pan‐Kla levels in Huh7‐WT cells treated with lenvatinib for indicated durations (months). (C) Representative CT images of HCC patients showing partial response (PR) and progressive disease (PD) to lenvatinib treatment. (D) Upper: AKR1B10 expression in PR/PD samples. Lower: AKR1B10 lactylation levels (anti‐AKR1B10 immunoprecipitation). (E) Pan‐Kla levels in anti‐AKR1B10 immunoprecipitates from Flag‐AKR1B10 HCC cells ± shAKR1B10. (Input controls: β‐actin (ACTB)/Flag). (F) Mass spectrometry identification of lactylation at AKR1B10 Lys173. (G) Pan‐Kla levels in anti‐Flag immunoprecipitates from HCC cells reconstituted with WT or K173R Flag‐AKR1B10. (Input controls: ACTB/Flag). (H) Dose‐response analysis of pan‐Kla levels in WT/K173R AKR1B10‐expressing HCC cells treated with Nala.(I) Time‐course analysis of pan‐Kla levels in WT/K173R AKR1B10‐expressing HCC cells treated with sodium lactate (Nala). (J‐K) AKR1B10 expression in WT or AKR1B10‐knockdown LR HCC cells reconstituted with graded doses of WT or K173R Flag‐AKR1B10. (Loading control: ACTB).

We next investigated the mechanisms underlying the markedly elevated AKR1B10 protein expression in LR cells. We first considered genomic alterations (Figure S4A). However, analysis of somatic copy number variations in the AKR1B10 gene from the TCGA cohort revealed no correlation between these events and AKR1B10 mRNA levels (Figure S4B). Since protein levels can also be modulated by PTMs,^41^ and given the association between LR, lactate and lactylation‐related gene signatures (Figure 1B), coupled with the known role of intracellular lactate in driving lysine lactylation (Kla),^42^ we hypothesized that lactylation might be activated in lenvatinib‐resistant HCC. Western blot analysis confirmed higher levels of pan‐lysine lactylation (pan‐Kla) in LR cell lines, compared to parental cells (Figure 3A). Pan‐Kla levels further increased with prolonged lenvatinib treatment (Figure 3B). We collected four specimens each from PR and PD patients (Figure 3C). Immunoprecipitation followed by WB using pan‐Kla antibodies detected both AKR1B10 and lactylated AKR1B10. Protein levels and lactylation levels of AKR1B10 were significantly higher in PD samples than in PR samples (Figure 3D). Consistently, AKR1B10 lactylation was also higher in LR cells than in parental cells (Figure S4C). Notably, shRNA‐mediated knockdown of AKR1B10 markedly reduced AKR1B10 lactylation levels in Flag‐AKR1B10‐expressing HCC cells (Figure 3E).

To identify specific lactylation sites, mass spectrometry analysis revealed a single lysine lactylation site at Lys173 (K173) within the AKR1B10 amino acid sequence (Figure 3F). K173 is highly conserved across species, from *Rattus norvegicus* to *Homo sapiens* (Figure S4D). We mutated AKR1B10 K173 to arginine (R) (AKR1B10 K173R) to mimic a non‐lactylatable state and expressed Flag‐tagged mutants (Flag‐AKR1B10 K173R) in HCC cells. Immunoprecipitation confirmed that lactylation of AKR1B10 was abolished by the K173R mutation (Figure 3G). Treatment with sodium L‐lactate (Nala) enhanced Flag‐AKR1B10 lactylation in a concentration‐dependent (5–20 mM, Figure 3H) and time‐dependent manner (Figure 3I). In contrast, Nala treatment did not increase lactylation of Flag‐AKR1B10 K173R (Figure 3H,I). We then explored whether lactylation modulates AKR1B10 protein expression. Unlike wild‐type Flag‐AKR1B10, Flag‐AKR1B10 K173R did not substantially increase AKR1B10 protein levels in HCC cells (Figure 3J). Furthermore, in LR HCC cells stably expressing shRNA against AKR1B10, wild‐type Flag‐AKR1B10, but not Flag‐AKR1B10 K173R, markedly increased AKR1B10 protein expression in a dose‐dependent manner (Figure 3K). Functionally, unlike wild‐type AKR1B10, Flag‐AKR1B10 K173R failed to enhance resistance to lenvatinib, showing no change in IC50 values or apoptosis rates (Figure S4E,F). As AKR1B10 is a key aldose reductase, we hypothesized that its lactylation status might influence HCC glucose metabolism through modulation of its enzymatic activity. To test this, we assessed AKR1B10 activity under varying lactylation levels. ELISA results indicated that wild‐type AKR1B10 overexpression enhanced its secretion; however, the K173R mutant failed to yield mature and stable protein (Figure S4G). By comparing the normalized enzyme activity, measured via NADPH consumption at 340 nm, in relation to AKR1B10 secretion levels in the supernatant, we found that the mutation at the K173 site did not significantly alter the enzyme activity of AKR1B10. These data indicate that AKR1B10 is lactylated at Lys173, which is critical for maintaining its protein stability and promoting the LR phenotype.

### 3.4 AARS1 interacts with and lactylates AKR1B10

**FIGURE 4:**
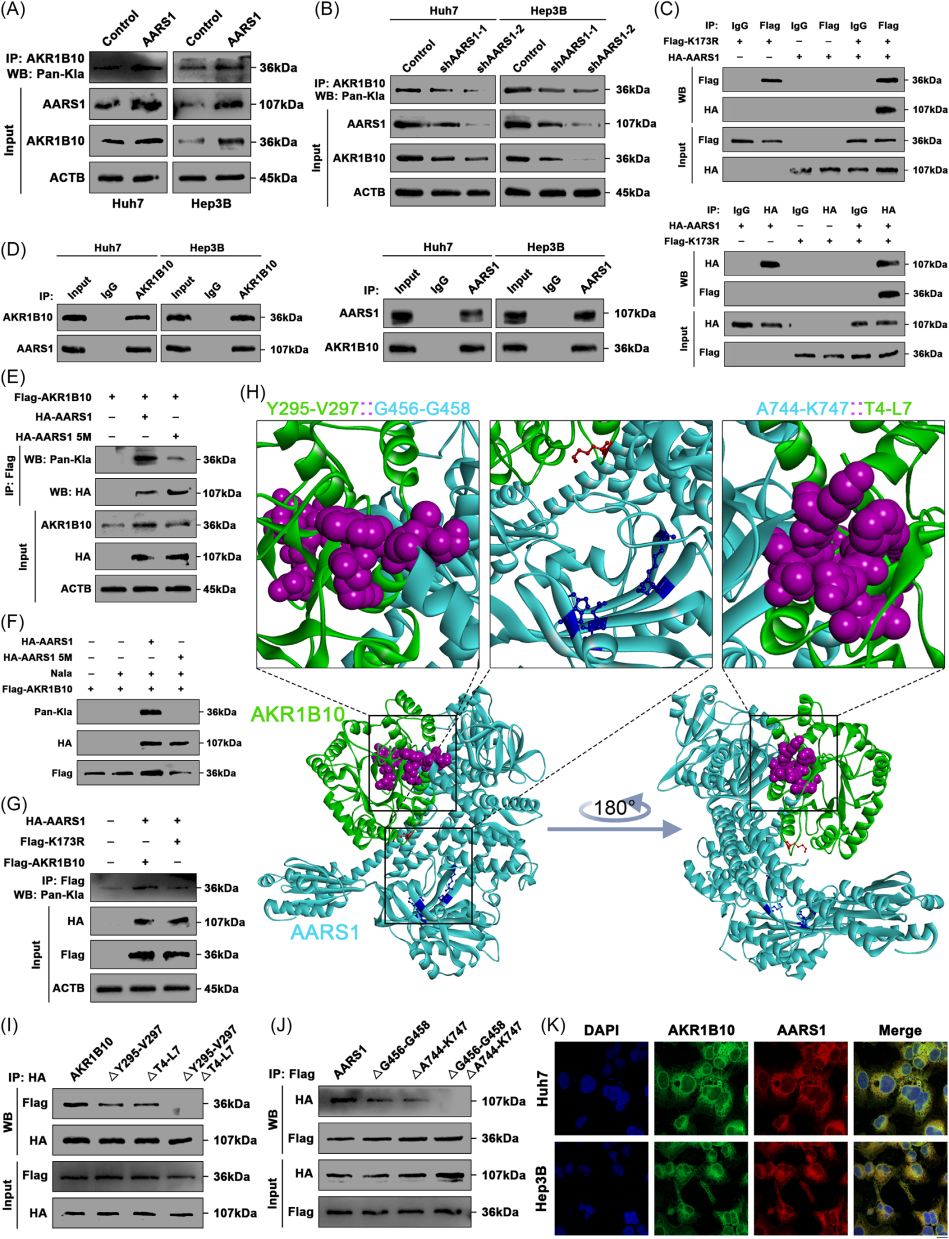
AARS1 interacts with and lactylates AKR1B10. (A) Co‐immunoprecipitation (Co‐IP) analysis of AKR1B10‐associated pan‐Kla, AARS1 and AKR1B10 in AARS1‐overexpressing HCC cells. (B) Co‐IP analysis of AKR1B10‐associated pan‐Kla, AARS1 and AKR1B10 in AARS1‐knockdown HCC cells. (C) Reciprocal Co‐IP of Flag‐AKR1B10 and HA‐AARS1 in 293T cells. Lysates immunoprecipitated with anti‐Flag or anti‐HA antibody. (Input controls: Flag/HA). (D) Endogenous AKR1B10‐AARS1 interaction in HCC cells. Lysates immunoprecipitated with indicated antibodies. (E) AKR1B10‐associated pan‐Kla levels in HCC cells overexpressing indicated constructs (Co‐IP). (F) Analysis of AARS1‐mediated AKR1B10 lactylation by reconstituting indicated vectors. (G) AKR1B10‐associated pan‐Kla levels in HCC cells overexpressing indicated mutant vectors (Co‐IP). (H) Predicted AKR1B10‐AARS1 binding interface modelled by Discovery Studio. (I) Co‐IP analysis of HA‐tagged WT/mutant AKR1B10 with Flag‐tagged AARS1 in 293T cells expressing indicated vectors. (J) Co‐IP analysis of Flag‐tagged WT/mutant AARS1 with HA‐tagged AKR1B10 in 293T cells expressing indicated vectors. (K) Immunofluorescence showing cytoplasmic co‐localization of AKR1B10 and AARS1 in HCC cells. Scale bars: 10 µm.

To identify the lactyltransferase responsible for catalyzing AKR1B10 lactylation using lactate and ATP, we screened several established lactyltransferases, including KAT5,^26^ KAT8,^43^ AARS1^44^ and AARS2.^45^ Correlation analysis of mRNA expression levels in the TCGA HCC cohort revealed that only AARS1 exhibited a strong positive correlation with AKR1B10 (Figure S5A,B). Co‐immunoprecipitation (Co‐IP) followed by WB demonstrated that overexpressing AARS1 increased both protein and lactylation levels of AKR1B10 in HCC cells (Figure 4A). Conversely, shRNA‐mediated suppression of AARS1 decreased AKR1B10 protein and lactylation levels (Figure 4B). Furthermore, siRNA depletion of the reported lactyltransferases KAT5 or KAT8 did not inhibit AKR1B10 Lys173 lactylation (Figure S5C), indicating that neither enzyme catalyses this specific modification. Survival analysis identified AARS1 as a significant risk factor for poorer OS, PFS and DFS in HCC patients (Figure S5D). Differential expression analysis showed higher AARS1 mRNA levels in most tumour types, including HCC, compared to adjacent normal tissues (Figure S5E). Spatial transcriptomics data from the HCCDB database confirmed relatively higher AARS1 expression in tumour regions versus para‐tumour regions (Figure S5F–H). Co‐IP experiments in 293T cells showed that reconstituted Flag‐AKR1B10 interacted directly with HA‐tagged AARS1. Flag‐AKR1B10 K173R also pulled down HA‐AARS1, indicating that the K173 mutation alone does not disrupt AKR1B10‐AARS1 binding (Figure 4C). Endogenous Co‐IP confirmed the association between AARS1 and AKR1B10 in HCC cells (Figure 4D). These results establish AARS1 as an AKR1B10‐interacting protein.

We next investigated whether AARS1 mediates AKR1B10 lactylation and stability. Based on reports that mutations in AARS1 catalytic pocket residues (R77A, M100A, W176E, V218D, D239A; denoted AARS1‐5 M) disrupt lactate binding,^44^ we constructed an HA‐tagged AARS1‐5 M mutant. Unlike wild‐type HA‐AARS1, HA‐AARS1‐5 M failed to enhance AKR1B10 lactylation (Figure 4E). Consistently, only wild‐type HA‐AARS1, not HA‐AARS1‐5 M, lactylated Flag‐AKR1B10 upon Nala treatment (Figure 4F). Moreover, while HA‐AARS1 increased lactylation in cells expressing Flag‐AKR1B10, Flag‐AKR1B10 K173R showed no such increase (Figure 4G), supporting the hypothesis that AARS1‐mediated lactylation at Lys173 is essential for AKR1B10 protein accumulation. To map the specific binding interfaces, molecular docking analysis predicted potential interaction sites: AKR1B10 residues Y295‐V297 with AARS1 residues G456‐G458, and AKR1B10 residues A744‐K747 with AARS1 residues T4‐L7 (Figure 4H). Notably, Lys173 of AKR1B10 is spatially proximal to the catalytic pocket of AARS1, positioning it favourably for lactylation catalysis. In vitro Co‐IP using various mutant constructs revealed that both predicted binding interfaces contribute independently to the AKR1B10‐AARS1 interaction (Figure 4I,J). Immunofluorescence confirmed the AARS1‐AKR1B10 interaction and their cytoplasmic colocalization (Figure 4K).

### 3.5 AARS1‐dependent Lys173 lactylation suppresses the K63 polyubiquitination‐mediated degradation of AKR1B10

**FIGURE 5:**
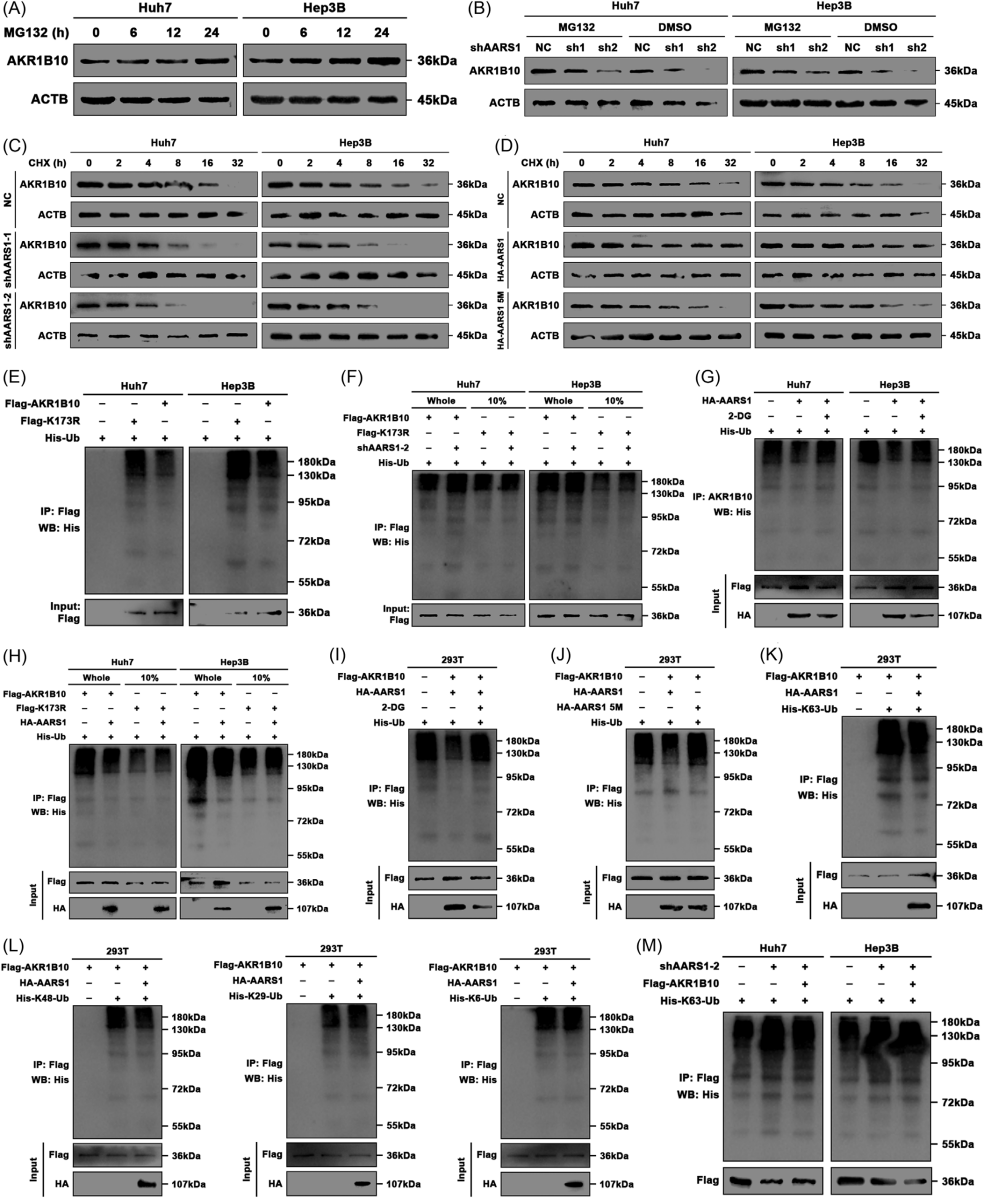
AARS1‐dependent Lys173 lactylation suppresses the K63 polyubiquitination‐mediated degradation of AKR1B10. (A) AKR1B10 protein levels in HCC cells treated with MG132 for indicated durations. (B) AKR1B10 levels in specified HCC cell lines transfected with shAARS1 ± MG132 treatment. (C) Effect of AARS1 knockdown on AKR1B10 half‐life in HCC cells treated with cycloheximide (CHX). (D) Effect of HA‐AARS1 versus catalytically inactive HA‐AARS1‐5 M on AKR1B10 half‐life (CHX chase). (E‐M) Anti‐His immunoblotting of anti‐Flag/anti‐AKR1B10 immunoprecipitates from transfected HCC/293T cells.

Lysine, an essential residue for protein structure and function, undergoes various PTMs.^41^ Notably, lysine lactylation has been reported to inhibit protein ubiquitination and subsequent proteasomal degradation.^46^ Therefore, we next investigated the mechanism by which lactylation of AKR1B10 at Lys173 regulates its protein level in HCC cells. First, we treated HCC cells with the proteasome inhibitor MG132. As anticipated, MG132 treatment increased endogenous AKR1B10 expression in a time‐dependent manner (Figure 5A). Moreover, MG132 effectively abolished the accelerated degradation of AKR1B10 induced by AARS1 silencing (Figure 5B). Furthermore, CHX chase assays demonstrated that silencing AARS1 significantly shortened the half‐life of the AKR1B10 protein (Figure 5C), whereas overexpression of HA‐AARS1, but not the catalytically inactive mutant HA‐AARS1 5 M, extended its half‐life (Figure 5D).

Based on data retrieved from the PhosphoSitePlus database, we noted that ubiquitination had previously been reported at Lys173 of AKR1B10 (Figure S6A).^47^ We then examined whether AARS1‐mediated lactylation of AKR1B10 modulates its ubiquitination. Using a ubiquitination ladder assay, we observed that the ubiquitination level of the AKR1B10 K173R mutant was markedly higher than that of wild‐type AKR1B10 (Figure 5E). Consistently, AARS1 silencing substantially increased the polyubiquitination level of wild‐type AKR1B10 but had no effect on the AKR1B10 K173R mutant (Figure 5F). Similarly, the polyubiquitination of endogenous AKR1B10 was significantly enhanced by the glycolysis inhibitor 2‐DG in HCC cells (Figure 5G). Conversely, ectopic expression of AARS1 markedly decreased the polyubiquitination of wild‐type AKR1B10, but not the K173R mutant (Figure 5H), and this decrease was reversed by 2‐DG (Figure 5I). In parallel, overexpression of wild‐type AARS1 (possessing lactyltransferase activity), but not HA‐AARS1 5 M, significantly suppressed AKR1B10 polyubiquitination (Figure 5J). Furthermore, AARS1 overexpression specifically inhibited K63‐linked polyubiquitination (Figure 5K) but not K6‐, K29‐ or K48‐linked polyubiquitination (Figure 5L) of AKR1B10. Conversely, AARS1 silencing significantly promoted K63‐linked polyubiquitination (Figure 5M).

We then subcutaneously injected athymic nude mice with Huh7 cells infected with lentivirus expressing either Flag‐tagged wild‐type AKR1B10 or the AKR1B10 K173R mutant. All tumours were treated with lenvatinib (Figure S6B). Consistent with the in vitro data, xenografts expressing wild‐type AKR1B10 exhibited significantly less sensitivity to lenvatinib, compared to those expressing the K173R mutant (Figure S6C,D). Immunohistochemical (IHC) staining of xenografts revealed strong AKR1B10 expression in tumours harbouring wild‐type AKR1B10, indicating that the K173R mutant failed to maintain AKR1B10 protein levels in vivo (Figure S6E). Collectively, these results demonstrate that lactylation at Lys173 prevents polyubiquitination‐mediated degradation of the AKR1B10 protein, thereby increasing its protein level.

### 3.6 Lys173‐lactylated AKR1B10 facilitates LDHA phosphorylation and increases the transcription level of LDHA by promoting histone lactylation

**FIGURE 6:**
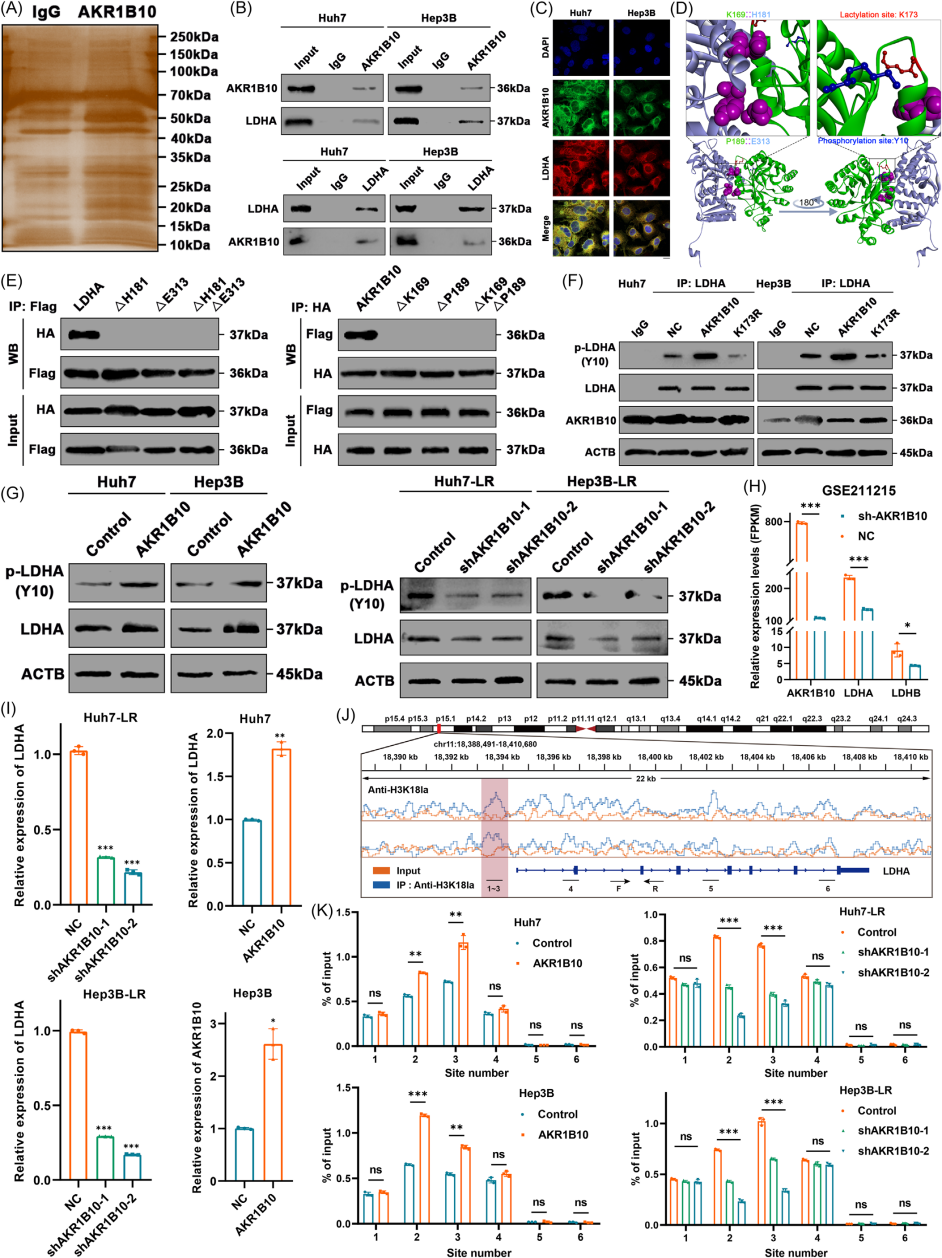
Lys173‐lactylated AKR1B10 facilitates LDHA phosphorylation and increases the transcription level of LDHA by promoting histone lactylation. (A) Silver staining of anti‐AKR1B10 immunoprecipitates from Huh7 cells. (B) Reciprocal Co‐IP of endogenous AKR1B10 and LDHA in HCC cells. (C) Immunofluorescence showing cytoplasmic co‐localization of AKR1B10 and LDHA. Scale bars: 10 µm. (D) Predicted AKR1B10‐LDHA binding interface modelled by Discovery Studio. (E) Co‐IP analysis of HA‐tagged AKR1B10 mutants with Flag‐tagged LDHA mutants in 293T cells. (F) p‐LDHA (Y10) and total LDHA levels in anti‐LDHA immunoprecipitates from HCC cells expressing indicated constructs. (Loading controls: AKR1B10/LDHA). (G) LDHA and p‐LDHA (Y10) expression in AKR1B10‐overexpressing HCC cells versus AKR1B10‐knockdown LR cells. (H) AKR1B10, LDHA and LDHB mRNA expression upon AKR1B10 suppression (GSE211215). (I) LDHA mRNA levels in AKR1B10‐overexpressing HCC cells versus AKR1B10‐knockdown LR cells. (J) IGV tracks of H3K18la enrichment at LDHA locus (ChIP‐seq, GSE156674). (K) ChIP‐qPCR quantification of H3K18la enrichment at LDHA promoter in AKR1B10‐modulated HCC cells. Data represent mean ± SD; *p < .05, **p < .01, ***p < .001, ns: not significant.

To further investigate the specific biological function of AKR1B10 in aerobic glycolysis, we performed mass spectrometry analysis of AKR1B10 immunoprecipitates from human HCC cells. This revealed that AKR1B10 interacts with LDHA and lactate dehydrogenase B (LDHB) (Figure 6A), well‐known enzymes catalyzing the conversion of pyruvate to lactate.^48^, ^49^ Analysis of scRNA‐seq data (GSE166635) surprisingly showed that LDHA mRNA expression was markedly higher in hepatocytes than LDHB, with LDHB exhibiting minimal expression in hepatocytes (Figure S7A). Spatial transcriptomics data from HCCDB further demonstrated that LDHA expression levels were significantly higher than LDHB within HCC cells, while LDHB was predominantly expressed in immune cells of the tumour microenvironment (Figure S7B). Therefore, LDHA was selected as the candidate interacting partner and effector molecule of AKR1B10 for subsequent studies. As expected, Co‐IP of endogenous proteins confirmed the association between AKR1B10 and LDHA in HCC cells (Figure 6B). Immunofluorescence assays corroborated this direct interaction, showing cytoplasmic colocalization of the two proteins (Figure 6C). To identify the specific binding sites, we modelled the AKR1B10‐LDHA interaction using Discovery Studio (Figure 6D). The predicted structure indicated potential binding sites between K169 of AKR1B10 and H181 of LDHA and between P189 of AKR1B10 and E313 of LDHA. In vitro Co‐IP experiments using mutant constructs further demonstrated that these specific residues on both AKR1B10 and LDHA are indispensable for their interaction (Figure 6E).

Given that LDHA enzymatic activity is regulated by phosphorylation at Tyr10 (Y10), which promotes tetramerization and enhances NADH binding,^50^ we noted the spatial proximity of AKR1B10 K173 to LDHA Y10 in the model (Figure 6D). This suggested that AKR1B10 K173 might modulate LDHA Y10 phosphorylation. Intriguingly, overexpression of wild‐type AKR1B10, but not the AKR1B10 K173R mutant, strongly enhanced LDHA Y10 phosphorylation (Figure 6F). Furthermore, AKR1B10 overexpression increased, while its suppression decreased, LDHA Y10 phosphorylation levels (Figure 6G). We observed that AKR1B10 expression levels also affected total LDHA protein levels, suggesting potential regulation of LDHA mRNA expression or protein stability by AKR1B10. Analysis of GSE211215 data showed that inhibition of AKR1B10 downregulated LDHA and LDHB mRNA levels (Figure 6H). Consistently, knocking down AKR1B10 in LR cells decreased LDHA mRNA, while overexpressing AKR1B10 in HCC cells increased it (Figure 6I). Given the high lactate levels in the tumour microenvironment, we hypothesized that LDHA mRNA upregulation might be mediated by histone lactylation as previously studied.^36^ However, mRNA stability assays indicated that AKR1B10 does not affect LDHA mRNA stability (Figure S7C). Re‐analysis of ChIP‐seq data (GSE156674) suggested that the LDHA promoter region might be regulated by histone H3 lysine 18 lactylation (H3K18la; Figure 6J). Supporting this, LDHA mRNA levels were downregulated by the glycolysis (and thus lactate production) inhibitor 2‐DG and upregulated by adding Rotenone (Figure S7D). ChIP‐qPCR assays using six primer pairs spanning the LDHA promoter confirmed significant enrichment of H3K18la at this locus (Figure 6J). Moreover, AKR1B10 suppression weakened, while stable AKR1B10 overexpression strengthened, H3K18la enrichment at the LDHA promoter (Figure 6K). These results establish that H3K18la positively regulates LDHA transcription.

### 3.7 The phosphorylation of the LDHA‐Y10 is crucial for AKR1B10‐mediated LR in HCC

**FIGURE 7:**
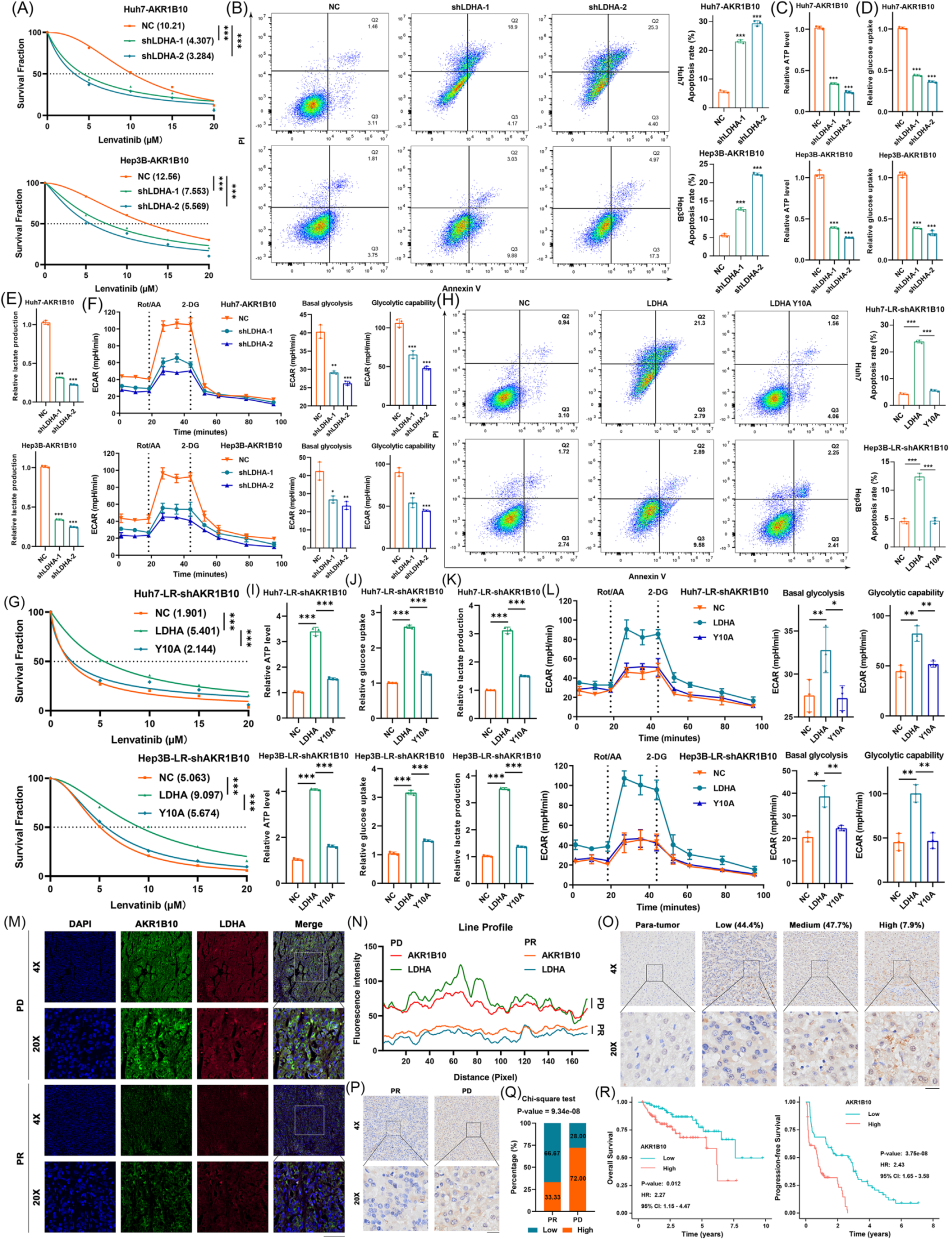
The phosphorylation of the LDHA‐Y10 is crucial for AKR1B10‐mediated LR in HCC. (A) Lenvatinib IC50 in HCC cells transfected with AKR1B10 ± LDHA knockdown. (B) Apoptosis rates in AKR1B10‐transfected HCC cells ± LDHA knockdown after lenvatinib treatment. (C–E) Intracellular ATP/glucose levels and extracellular lactate production in AKR1B10‐transfected HCC cells ± LDHA knockdown. (F) ECAR profiles (basal glycolysis/glycolytic capacity) in above cells. (G‐H) Lenvatinib IC50 and apoptosis rates in AKR1B10‐knockdown HCC cells reconstituted with LDHA‐WT or phosphorylation LDHA‐Y10A. (I–K) Intracellular ATP/glucose levels and extracellular lactate production in AKR1B10‐knockdown cells reconstituted with LDHA‐WT/Y10A. (L) ECAR profiles (basal glycolysis/glycolytic capacity) in above cells. (M) Confocal images of LDHA (red)/AKR1B10 (green) co‐localization in PR/PD patient samples. Nuclei: DAPI (blue). Scale bar: 50 µm. (N) Co‐localization quantified by ImageJ. Fluorescence intensity analysis. (O) Representative immunohistochemistry (IHC) of AKR1B10 in HCC/adjacent tissues (low/medium/high expression). Scale bar: 20 µm. (P) IHC of AKR1B10 in PR/PD samples. Scale bar: 20 µm. (Q) AKR1B10‐positive rates in PR versus PD samples. (R) Survival analysis by AKR1B10 IHC status (OS/PFS). Data: mean ± SD; *p < .05, **p < .01, ***p < .001.

To functionally determine the necessity of LDHA Y10 phosphorylation for AKR1B10, we performed rescue experiments modulating LDHA expression and phosphorylation in specific HCC cell models. First, we knocked down LDHA expression using shRNAs in HCC cells stably overexpressing AKR1B10. qRT‐PCR and WB confirmed efficient LDHA silencing, resulting in decreased LDHA protein and LDHA Y10 phosphorylation levels (Figure S8A,B). Notably, LDHA knockdown also reduced AKR1B10 expression, suggesting a potential positive feedback loop between these proteins (Figure S8B). As expected, suppressing LDHA significantly increased lenvatinib sensitivity in AKR1B10‐overexpressing cells, evidenced by a decreased IC50 value and increased apoptosis (Figure 7A,B). Consistently, LDHA knockdown attenuated the AKR1B10‐mediated enhancement of aerobic glycolysis, reducing cellular ATP levels, glucose uptake, extracellular lactate production (Figure 7C–E), and ECAR, including basal glycolysis and glycolytic capacity (Figure 7F). Next, in LR cells with stable AKR1B10 suppression, we reintroduced either wild‐type LDHA or the phosphorylation‐defective mutant LDHA Y10A. WB verified successful expression (Figure S8C). Unlike wild‐type LDHA, re‐expression of LDHA Y10A failed to rescue LR, showing no significant changes in IC50 values or apoptosis rates (Figure 7G,H). Functionally, wild‐type LDHA restored cellular ATP levels, glucose uptake and lactate production, whereas LDHA Y10A had no effect, demonstrating that Y10 phosphorylation is essential for AKR1B10's pro‐glycolytic effects (Figure 7I–K). Seahorse analysis confirmed that LDHA Y10A overexpression failed to restore ECAR, basal glycolysis or glycolytic capacity in AKR1B10‐knockdown LR cells (Figure 7L). These data indicate that phosphorylation of LDHA at Y10 is essential for maintaining its enzymatic activity in aerobic glycolysis and promoting the AKR1B10‐mediated LR phenotype.

To assess the clinical relevance of the AKR1B10‐LDHA axis in LR, we analysed their expression and colocalization in post‐treatment surgical HCC samples. Immunofluorescence staining revealed that patients with low AKR1B10 and LDHA levels responded better to lenvatinib (exhibiting greater reductions in fluorescence intensity), compared to those with high expression (Figure 7M,N). Both proteins primarily colocalized in the cytoplasm. To further evaluate AKR1B10 as a potential HCC biomarker, immunohistochemical staining was performed on 161 HCC specimens. Samples were categorized into low, medium or high AKR1B10 expression based on immunoreactivity, and AKR1B10 expression was significantly lower in adjacent non‐tumour tissues than in HCC tissues (Figure 7O). Furthermore, tissues from patients with PD exhibited higher AKR1B10 expression than those from patients with PR (Figure 7P,Q). Survival analysis demonstrated that patients with higher AKR1B10 protein levels had significantly shorter OS and PFS, suggesting AKR1B10 may serve as both a predictor of lenvatinib response and a prognostic risk factor (Figure 7R).

### 3.8 Targeting AKR1B10‐LDHA axis increases lenvatinib efficacy in HCC

**FIGURE 8:**
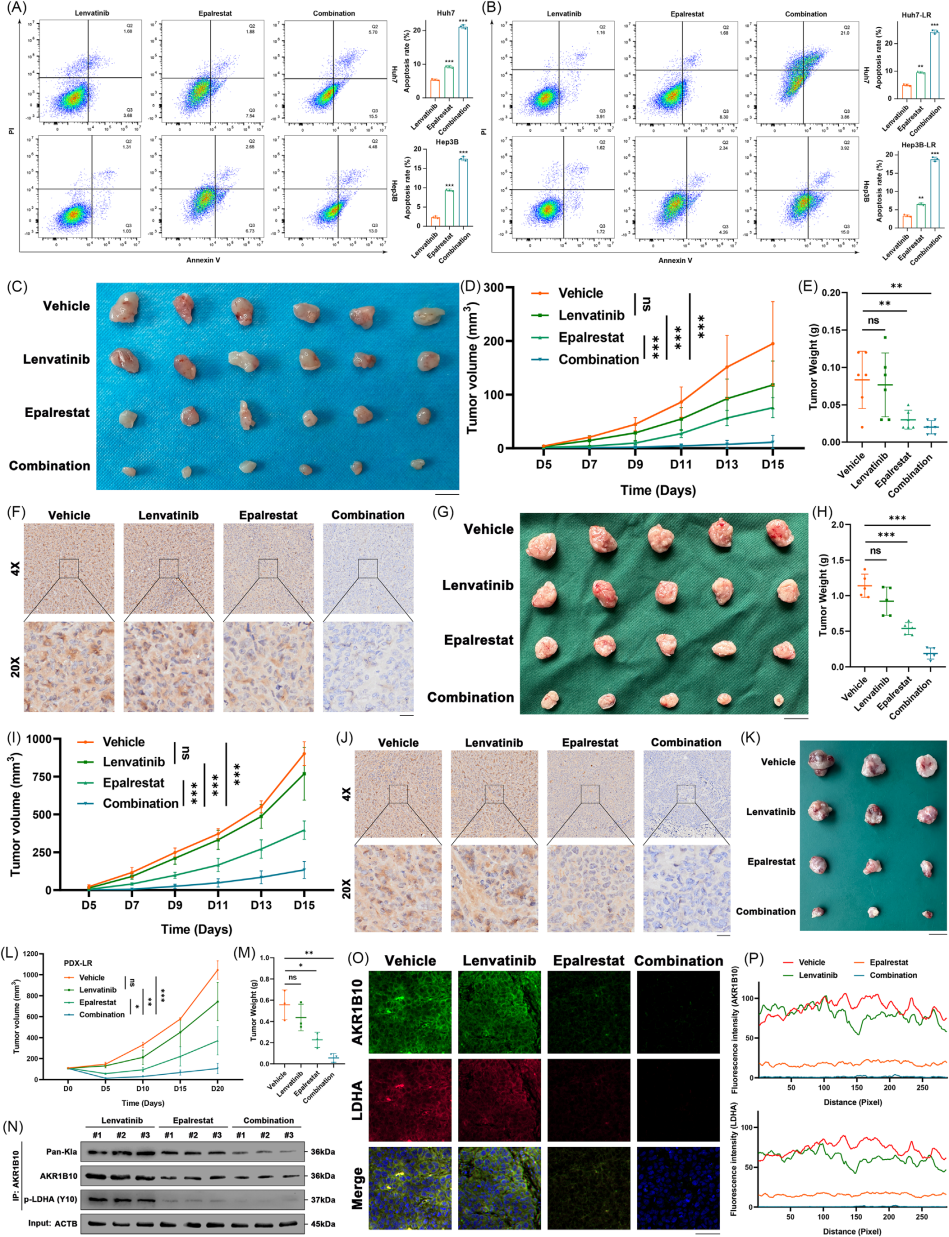
Targeting AKR1B10–LDHA axis increases lenvatinib efficacy in HCC. (A‐B) Apoptosis rates in HCC/LR cells treated with EPA, lenvatinib or combination. (C–E) Tumour volumes/growth curves/weights in mice bearing LR‐Huh7 xenografts (*n* = 6/group). (F) IHC of AKR1B10 in xenograft groups. Scale bar: 20 µm. (G–I) Tumour volumes/growth curves/weights in mice bearing LR‐Hepa1‐6 xenografts (*n* = 5/group). (J) IHC of AKR1B10 in Hepa1‐6 xenografts. Scale bar: 20 µm. (K–M) Tumour volumes/growth curves/weights in patient‐derived xenograft (PDX) models (*n* = 3/group). (N) AKR1B10 lactylation, total AKR1B10 and p‐LDHA(Y10) levels in anti‐AKR1B10 immunoprecipitates from xenografts. (O) LDHA (red)/AKR1B10 (green) co‐localization in xenografts. Nuclei: DAPI (blue). Scale bar: 50 µm. (P) Co‐localization quantified by ImageJ. Data: mean ± SE; *p < .05, **p < .01, ***p < .001; ns: not significant.

As previously reported, EPA is an inhibitor of AKR1B10, suppressing its secretion and enzymatic activity in multiple cancer types.^51^ Therefore, this study aimed to verify EPA's inhibitory effect on AKR1B10 and explore its potential synergistic anticancer effect with lenvatinib in HCC. In vitro, EPA combined with lenvatinib induced significantly higher apoptosis rates than lenvatinib monotherapy in both parental HCC cells and LR cells (Figure 8A,B), suggesting a synergistic anticancer effect. To further evaluate the therapeutic synergy in vivo, we subcutaneously inoculated LR Huh7 cells into athymic nude mice. Tumours treated simultaneously with EPA and lenvatinib exhibited significantly better responses, compared to either monotherapy alone (Figure 8C). Consistent with this, combined therapy resulted in significantly greater reductions in tumour weight and volume from baseline in individual mice, compared to monotherapy groups (Figure 8D,E). Immunohistochemical analysis of these tumours confirmed that the combination therapy significantly reduced the expression of both AKR1B10 and LDHA (Figures 8F and S9A).

To corroborate these findings, we established a lenvatinib‐resistant mouse Hepa1‐6 (Hepa1‐6 LR) cell line. Validation included higher IC50 values for lenvatinib and increased pan‐Kla levels, compared to parental cells (Figure S9B,C). WB and immunoprecipitation analyses further showed upregulation of AKR1B10, LDHA, LDHA Y10 phosphorylation and AKR1B10 lactylation in Hepa1‐6 LR cells (Figure S9D,E). Mirroring the Huh7 model, the EPA–lenvatinib combination elicited significantly superior antitumour responses in mice bearing Hepa1‐6 LR tumours, compared to monotherapies (Figure 8G), accompanied by significantly greater reductions in tumour weight and volume (Figure 8H,I). IHC again demonstrated significantly lower AKR1B10 and LDHA expression in the combination group (Figures 8J and S9F).

To further investigate the role of AKR1B10 in LR and the therapeutic potential, we utilized PDX models established from lenvatinib‐resistant HCC patients. PDX models treated with the EPA–lenvatinib combination developed significantly smaller and lighter tumours than those treated with vehicle, EPA alone or lenvatinib alone (Figure 8K–M). Moreover, mice bearing tumours received monotherapy of EPA or combined therapy did not show any body weight loss, while this effect was observed in mice tumours without treatment or lenvatinib alone (Figure S9G). Remarkably, mice treated with monotherapy or combined therapy did not exhibit hepatic or renal side effects (Figure S9H). Consistently, WB and immunoprecipitation analyses indicated that the combination therapy significantly suppressed AKR1B10 lactylation and LDHA Y10 phosphorylation, compared to monotherapies (Figure 8N). Immunofluorescence staining confirmed the downregulation of both AKR1B10 and LDHA protein levels in the combination group within the LR PDX tumours (Figure 8O,P). Collectively, these results demonstrate that combined therapy with EPA and lenvatinib exerts synergistic effects in overcoming LR in HCC.

As EPA is a well‐known inhibitor for multiple aldehyde reductases, to further validate the crucial role of targeting AKR1B10 in the synergistic treatment with lenvatinib, we directly employed a genetic knockout approach using a CRISPR/Cas9‐sgRNA system. It confirmed the outstanding knockout efficiency of AKR1B10 sgRNA using WB tests in Huh7 and Hep3B LR cells (Figure S10A). As expected, AKR1B10 knockout significantly enhanced lenvatinib sensitivity, evidenced by reduced IC50 values and increased apoptosis in LR cells (Figure S10B,C). Consistent with this, AKR1B10 knockout in LR cells decreased cellular ATP levels, glucose uptake and extracellular lactate levels, indicating inhibition of aerobic glycolysis (Figure S10D,E). Seahorse analysis further revealed decreased ECAR, including basal glycolysis and glycolytic capacity, upon AKR1B10 knockout in LR cells (Figure S10G). In vivo mouse models using Huh7 LR cells, the AKR1B10 knockout alone exhibited a significant tumour‐suppressive effect and, critically, synergized with lenvatinib to a comparable degree as EPA combined with Lenvatinib (Figure S10H,J). The fact that both knockout of AKR1B10 and pharmacological inhibition with EPA produce virtually identical therapeutic outcomes—both as monotherapy and in combination with lenvatinib—provides a powerful and convergent line of evidence.

## 4 DISCUSSION

Our study proposes a model in which AKR1B10 drives LR in HCC. AKR1B10 undergoes AARS1‐mediated lactylation at Lys173, which enhances its protein stability by antagonizing ubiquitination. This stabilization enables AKR1B10 to facilitate phosphorylation of LDHA at Y10 and promote LDHA transcription through H3K18la. The resulting increase in LDHA expression and activity further amplifies lactate production via enhanced aerobic glycolysis. This creates a regenerative feedback loop that sustains LR.

The expanding role of protein lactylation, affecting both histone and non‐histone proteins, signifies a paradigm shift in understanding cancer metabolism and epigenetic reprogramming. Lactate, once considered a metabolic waste product, is now recognized as a critical substrate for lactylation modifications that drive oncogenic signaling, metastasis and therapy resistance. For instance, ABCF1 lactylation at K430 induces its nuclear translocation, enabling binding to the KDM3A promoter and activation of the KDM3A–H3K9me2–HIF1α axis, enhancing glycolysis and lactate production in HCC.^52^ Similarly, hypoxia‐inducible factor‐1 alpha (HIF‐1α) lactylation stabilizes this transcription factor in prostate cancer, amplifying angiogenesis and metabolic adaptation.^53^ In breast cancer, KAT2A‐mediated RCC2 lactylation at K124 facilitates RCC2 recruitment of SERBP1, stabilizing MAD2L1 mRNA and promoting progression.^54^ While AKR1B10's involvement in LR was previously unreported, our work not only establishes its role in regulating aerobic glycolysis but also identifies Lys173 as its specific lactylation site. Although we demonstrate that lactylation stabilizes AKR1B10 by competing with ubiquitination at Lys173, the potential impact of this modification on AKR1B10's secretory function remains unexplored. Lactylation could potentially modulate secretion by altering protein conformation, stability or solubility. Given that AKR1B10 is a validated secretory protein detectable by commercial ELISA kits, and its secreted levels serve as a prognostic indicator in liver cancer,^10^, ^16^ investigating the effect of lactylation on its secretion warrants future research. Prospective studies incorporating serial serum AKR1B10 measurements via ELISA are warranted to further validate its utility as a dynamic biomarker for predicting lenvatinib treatment response. Lack of detecting global lactoylation modification of proteins between LR and parental HCC cells is also a limitation for this study. Future studies employing global lactylome profiling could uncover additional lower‐abundance lactylation sites on AKR1B10 and other proteins, further expanding our understanding of the lactylation network.

LDHA, a pivotal glycolytic enzyme, is tightly regulated at transcriptional and post‐translational levels. Transcriptionally, factors like cellular Myc proto‐oncogene (c‐Myc),^55^ hypoxia‐inducible factor 1 (HIF1),^56^ cAMP response element‐binding protein (CREB),^57^ and activator protein‐1 (AP‐1)^58^ regulate LDHA in various cancers. Furthermore, histone lactylation, fuelled by abundant lactate (LDHA's own enzymatic product), establishes a positive feedback loop for LDHA transcription, as identified in our study. Post‐translationally, key modifications profoundly impact LDHA activity. Phosphorylation, notably at Y10, potently activates LDHA, driving processes like invasion and anoikis resistance in breast cancer.^59^ Other regulators include hCINAP, facilitating FGFR1‐mediated Y10 phosphorylation to sustain colorectal cancer stemness; ^60^ ULK1, enhancing LDHA activity at S196 under nutrient stress to boost lactate and promote autophagy via Vps34 lactylation; ^29^ and TTK kinase, phosphorylating Y239 in PDAC to form a feed‐forward loop amplifying glycolysis and H3K18la.^61^ Conversely, lysine acetylation inversely regulates LDHA: K5 deacetylation by SIRT2 activates and stabilizes it in pancreatic cancer, while K5 acetylation triggers lysosomal degradation.^62^ Additionally, FKBP10 promotes clear cell renal cell carcinoma progression and modulates HIF2α blockade sensitivity by facilitating LDHA phosphorylation.^30^ Our findings reveal a novel regulatory axis where LDHA is activated by AKR1B10, highlighting critical enzyme–enzyme crosstalk in cancer metabolism. However, in the current study, the specific mechanism by which AKR1B10 promotes the phosphorylation of LDHA and the specific types of kinases that mediate this phosphorylation modification have not been thoroughly explored. We just stated that AKR1B10 enhances LDHA Y10 phosphorylation, likely by facilitating the recruitment of an unidentified upstream tyrosine kinase, rather than acting as a kinase itself. We deeply acknowledge this limitation and plan to pursue this line of inquiry in our immediate future work.

EPA, an aldose reductase inhibitor, is not yet clinically established for HCC treatment. However, preclinical evidence suggests its potential to sensitize tumours to lenvatinib by targeting metabolic vulnerabilities. LR can involve compensatory EGFR upregulation, activating survival pathways like PAK2‐ERK5 and MEK‐ERK.^6^ Although EPA is not an EGFR inhibitor, its ability to disrupt aerobic glycolysis and lactate flux may synergize with lenvatinib's anti‐angiogenic effects by targeting the energy metabolism essential for resistant cells. Despite the lack of HCC‐specific clinical data for EPA, its mechanism aligns with strategies to overcome LR. Future studies should validate if EPA enhances lenvatinib efficacy by disrupting glycolytic metabolism in HCC. Importantly, this study mainly elaborates that AKR1B10 drives LR in HCC via AARS1‐mediated lactylation‐stabilization, initiating a lactate/H3K18la positive feedback loop that hyperactivates glycolysis; targetable by EPA–lenvatinib synergy.

## 5 CONCLUSION

In conclusion, this study elucidates the critical role of AKR1B10 in mediating LR in HCC via a glycolytic feed‐forward loop. Bioinformatic analysis and validation in LR cell lines identified AKR1B10 as a key resistance driver. Functional assays demonstrated its dependence on aerobic glycolysis to confer resistance. Crucially, AKR1B10 undergoes AARS1‐catalysed lactylation at Lys173, stabilizing the protein by antagonizing ubiquitination. Stabilized AKR1B10 interacts with and enhances LDHA phosphorylation at Y10, while simultaneously, AKR1B10‐generated lactate fuels H3K18la to transcriptionally upregulate LDHA. Significantly, targeting AKR1B10 with EPA synergizes with lenvatinib to overcome resistance, highlighting a promising therapeutic strategy.

## AUTHOR CONTRIBUTIONS

**Zijian Liu**: Conceptualization; data curation; formal analysis; funding acquisition; investigation; software; validation; visualization; writing—original draft. **Jingsheng Yuan**: Conceptualization; data curation; funding acquisition; investigation; validation. **Shitong Su**: Data curation; investigation; software; writing—review and editing. **Jiaqi Han**: Funding acquisition; investigation; software; validation; visualization; writing—original draft. **Ni Zeng**: Validation; visualization. **Yuhan Ma**: Validation; visualization. **Nianyong Chen**: Conceptualization; supervision. **Tao Lv**: Conceptualization; funding acquisition; project administration; supervision; writing—review and editing.

## CONFLICT OF INTEREST STATEMENT

The authors declare no conflicts of interest.

## ETHICS STATEMENT

The study using clinical samples was approved by the Ethics Committee on Biomedical Research, West China Hospital of Sichuan University (2016, no. 120). Informed consent was obtained from all patients or their relatives. All operations of experimental animals were performed in accordance with the National Institutes of Health's Guide for the Care and Use of Laboratory Animals. All operations were approved by the Animal Care and Use Committee of West China Hospital of Sichuan University (2020351A).

## Supporting information



Supporting Information

## Data Availability

All data included in this study are available upon request by contact with the corresponding author.
