# Supplementary material for: AARS1‐mediated AKR1B10 lactylation stabilizes an aerobic glycolysis‐positive feedback loop to drive lenvatinib resistance in hepatocellular carcinoma
Source: Clin Transl Med. 2025 Dec 26;16(1):e70561. doi: 10.1002/ctm2.70561 (PMC12743142; doi:10.1002/ctm2.70561)
Supplement: Supplementary file 1 — Supporting Information [file CTM2-16-e70561-s001.docx]

**Supplementary Figures**

**
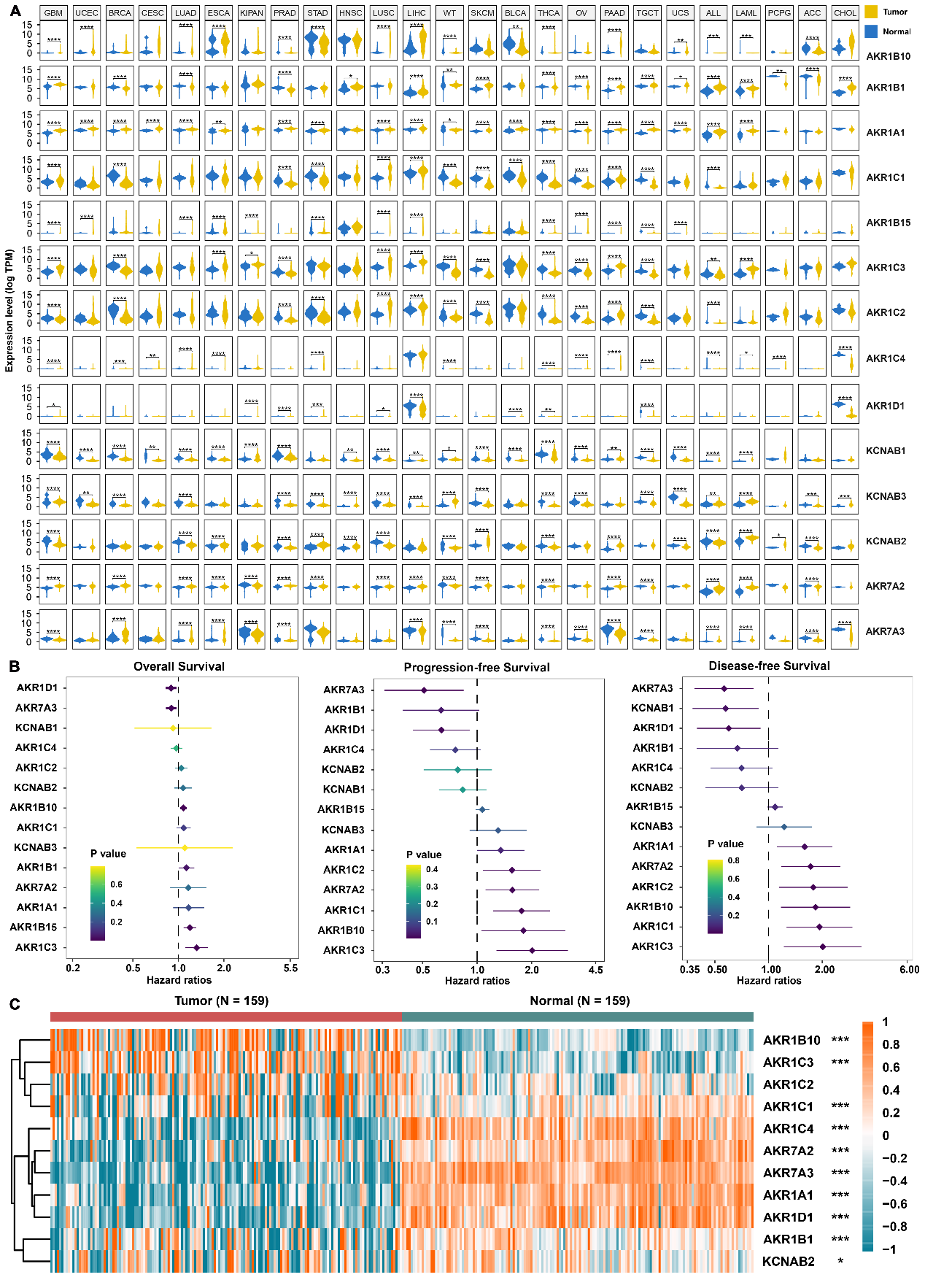
**

**Figure S1. I****ntegrated analysis of AKRs with multi-omics data.** (A) Transcriptional levels of fourteen AKRs in 25 tumor types compared to normal tissues in GTEx and TCGA datasets. (B) Univariate Cox analysis of AKRs for overall survival (OS), progression-free survival (PFS), and disease-free survival (DFS) in TCGA. (C) Protein levels of AKRs in HCC tumor tissues and para-tumor tissues in public proteomic data. The asterisks represent the statistical p-value (* p < 0.05, ** p < 0.01, *** p < 0.001, **** p < 0.0001, no lines, no significance).


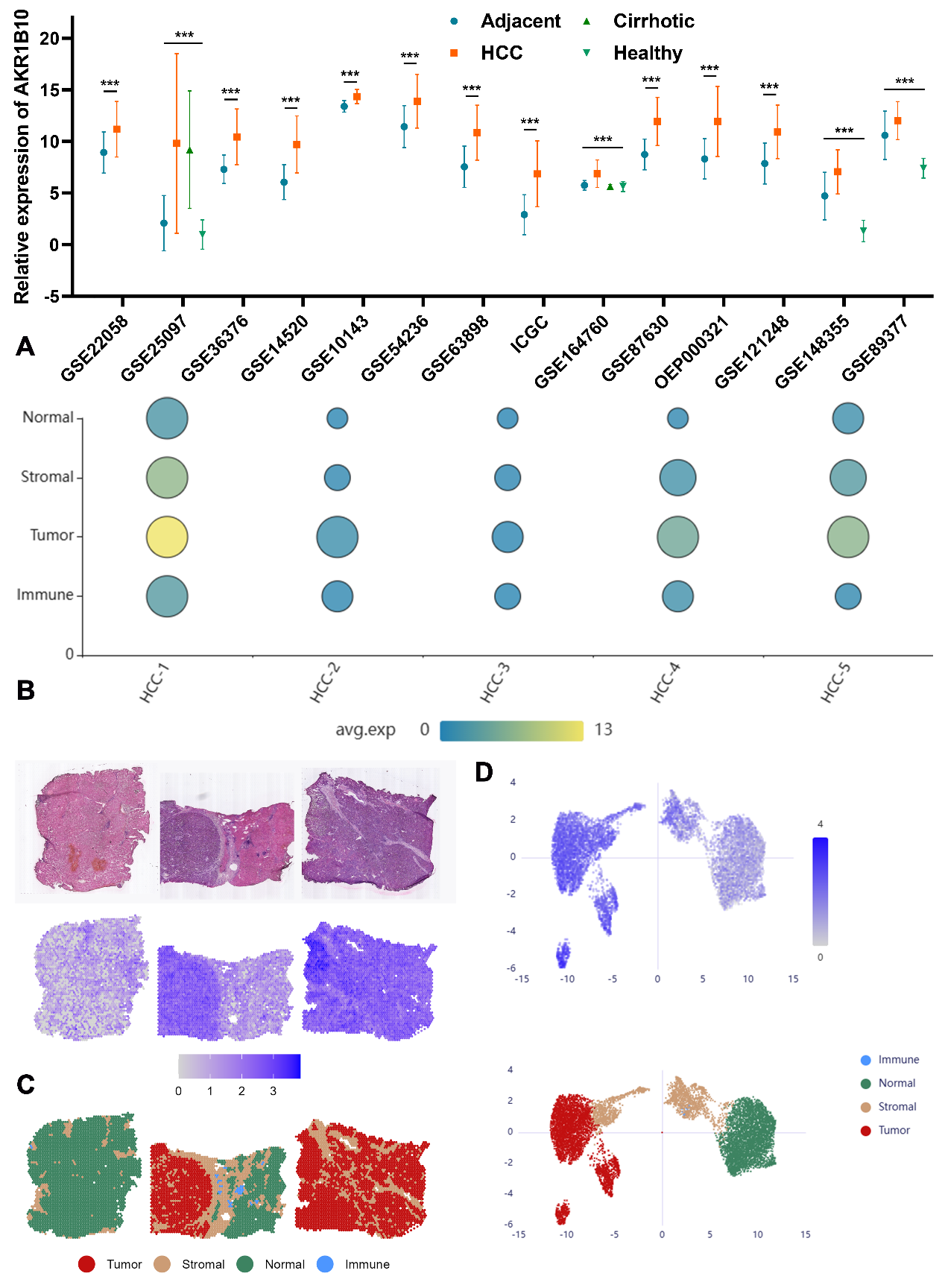


**Figure S2. Single-cell transcriptome and spatial transcriptomics data of AKR1B10 in HCCDB database.** (A) The mRNA expression level of AKR1B10 in HCC compared with adjacent tissues across fourteen public datasets in HCCDB database. (B-D) The distribution of AKR1B10 in different IHC area in spatial transcriptomics data. The asterisks represent the statistical p-value (** p < 0.01, *** p < 0.001).


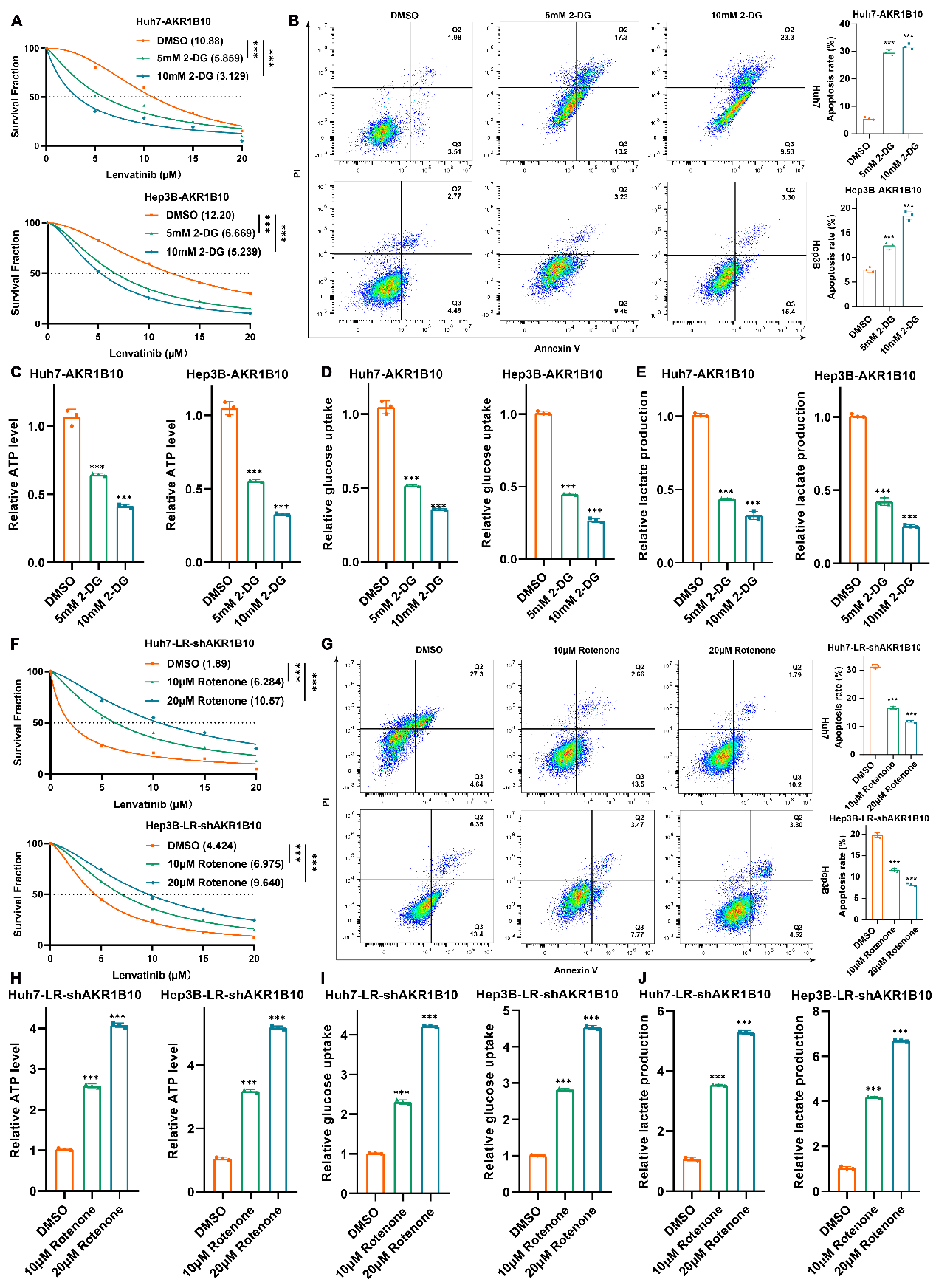


**Figure S3. AKR1B10 mediates lenvatinib resistance depending on aerobic glycolysis.** (A) IC50 values for HCC cells that stably overexpression of AKR1B10 when treated with lenvatinib plus 2-DG. (B) Apoptosis rates for HCC cells that stably overexpression of AKR1B10 when treated with lenvatinib plus 2-DG. (C-E) Intracellular ATP levels, intracellular glucose levels and relative lactate production in HCC cells that stably overexpression of AKR1B10 when treated with 2-DG. (F) IC50 values for HCC cells that stably suppression of AKR1B10 when treated with lenvatinib plus Rotenone. (G) Apoptosis rates for HCC cells that stably suppression of AKR1B10 when treated with lenvatinib plus Rotenone. (H-I) Intracellular ATP levels, intracellular glucose levels and relative lactate production in HCC cells that stably suppression of AKR1B10 when treated with Rotenone. The asterisks represent the statistical p-value (*** p < 0.001).


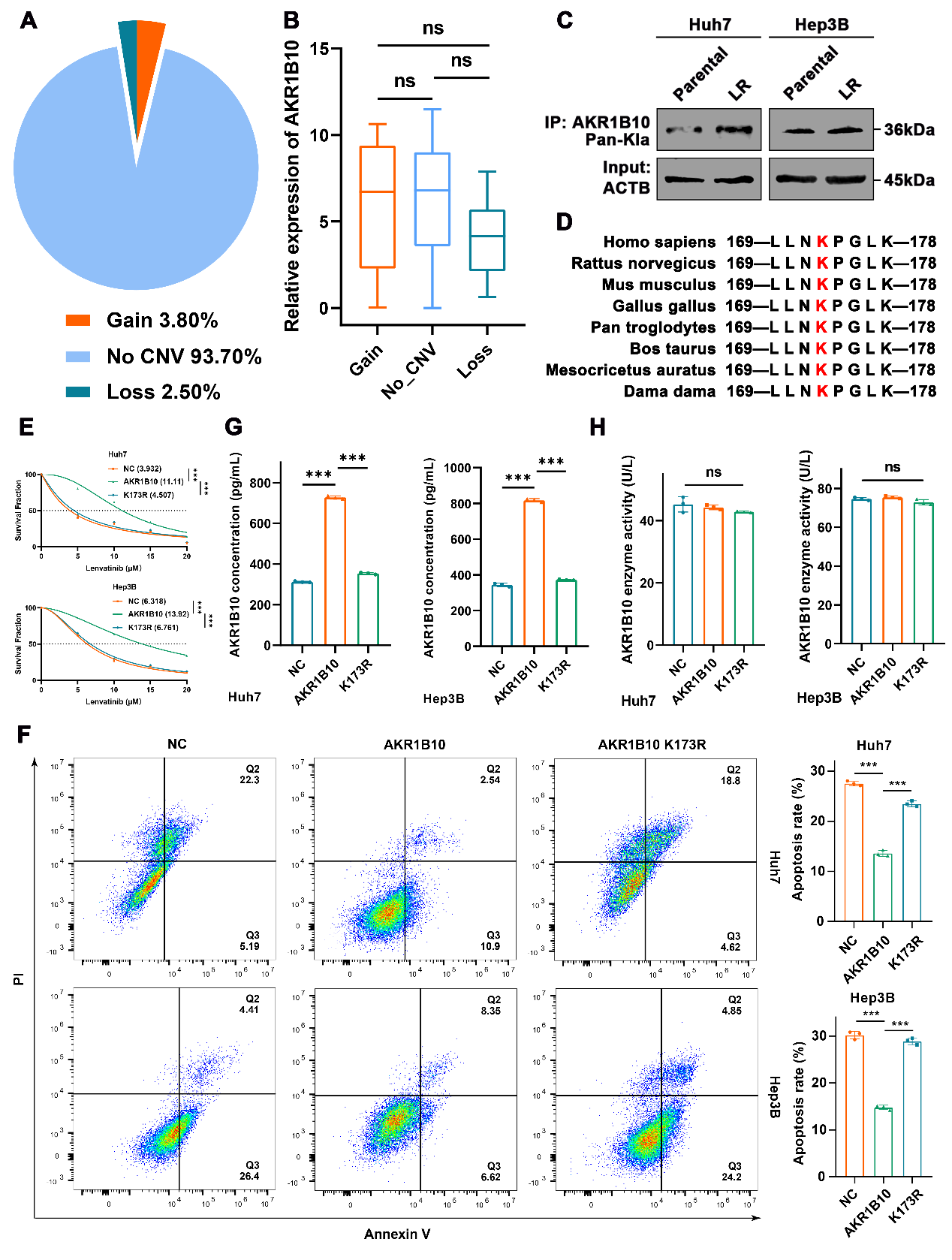


**Figure S4. Lactylation at Lys173 maintains the biological function of AKR1B10. (A)** Somatic copy number variations (CNV) of AKR1B10 in HCC samples from TCGA HCC cohort. (B) The relative mRNA expression levels of AKR1B10 in different CNV groups in HCC cohort. (C) Western blot analysis comparing lactylation levels of AKR1B10 in parental and LR HCC cells. (D) Lys 173 is highly conserved in different species, ranging from Rattus norvegicus to Homo sapiens. (E) IC50 values for HCC cells re-expression of WT Flag-AKR1B10 and Flag-AKR1B10 K173R when treated with lenvatinib. (F) Apoptosis rates for HCC cells reconstituted expression of WT Flag-AKR1B10 and Flag-AKR1B10 K173R when treated with lenvatinib. (G) Concentration levels of AKR1B10 in indicated HCC cells tested by ELISA kit. (H) The enzyme activity levels of AKR1B10 in indicated HCC cells evaluated by monitoring the decline in absorbance of NADPH at 340 nm. The asterisks represent the statistical p-value (*** p < 0.001, ns, no significance).

**
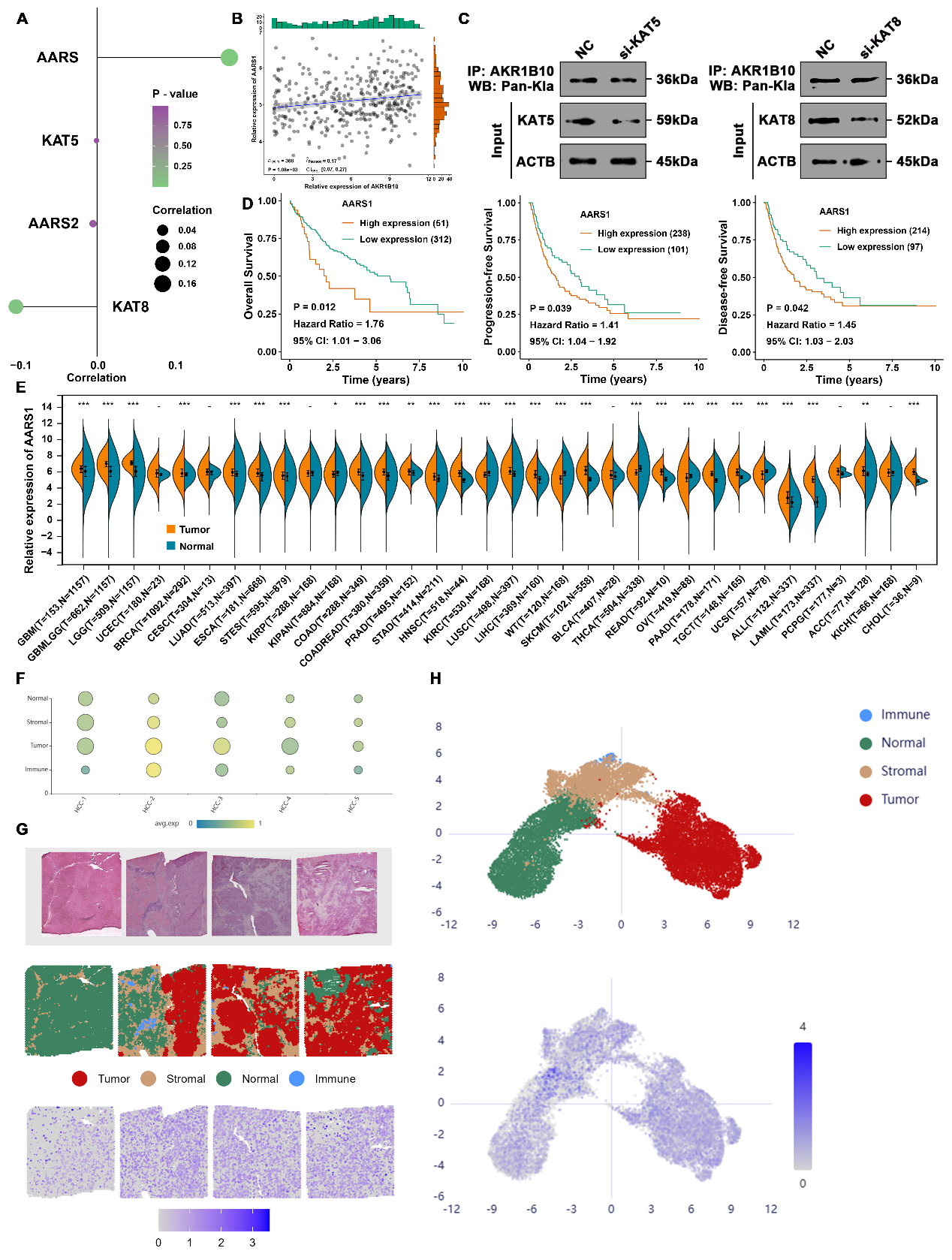
**

**Figure S5. Integrated analysis of AARS1 with multi-omics data.** (A) Correlation analysis among the expression levels of AKR1B10 and several lactyltransferases in TCGA HCC data. (B) Correlation analysis among the expression levels of AKR1B10 and AARS1 in TCGA HCC data. (C) Representative western blot analysis of Pan-Kla following immunoprecipitation with AKR1B10 in Huh7 cell transfected with siKAT5 or siKAT8. (D) Survival analysis of AARS1 in terms of OS, PFS and DFS for HCC patients. (E) Transcriptional levels of AARS1 in Pan-cancers compared to normal tissues in GTEx and TCGA datasets. (F-H) The distribution of AARS1 in different IHC area in spatial transcriptomics data. The asterisks represent the statistical p-value (* p < 0.05, ** p < 0.01, *** p < 0.001, -, no significance).


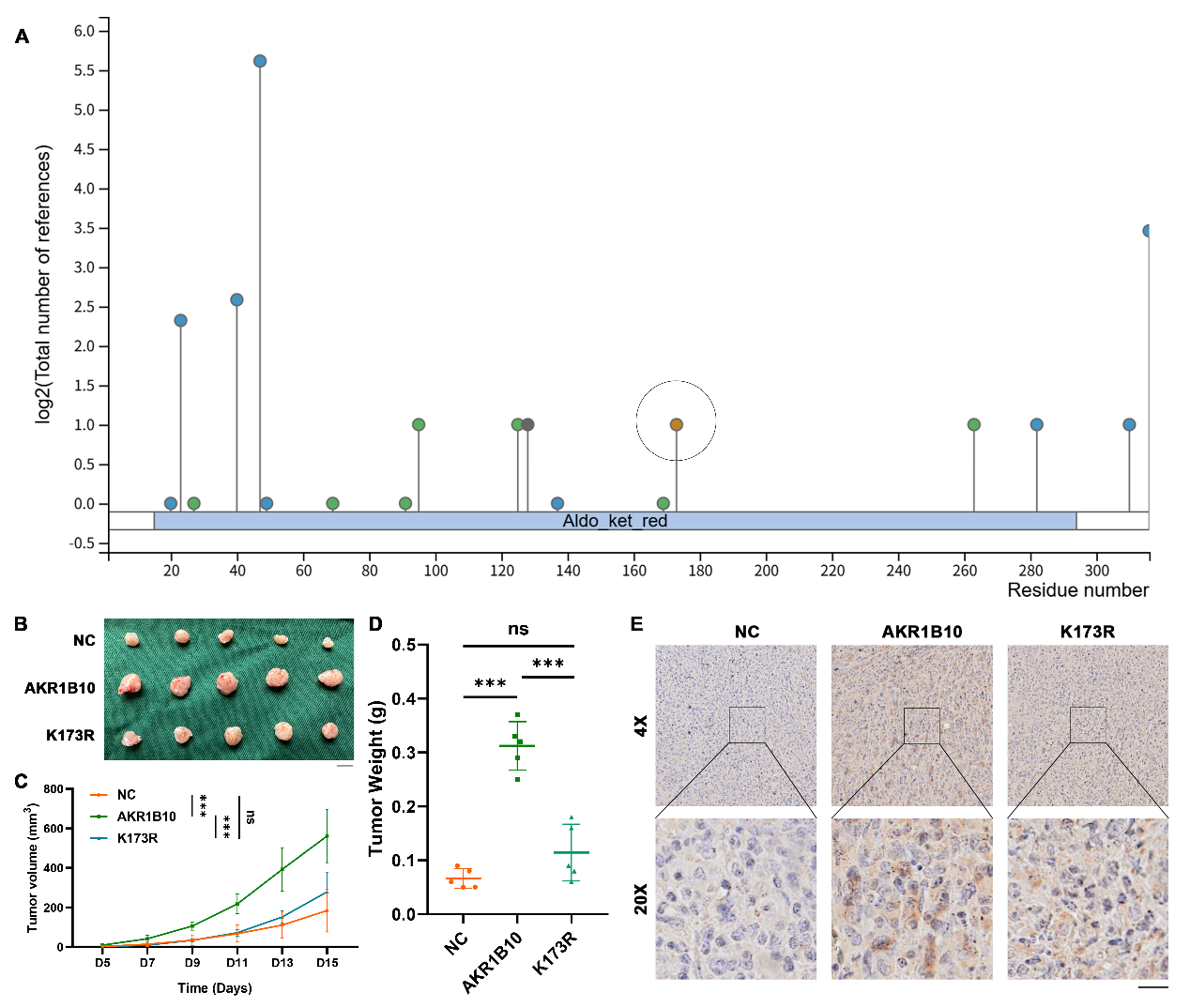


**Figure S6. Lactylation at Lys173 enhances AKR1B10 protein expression in vivo.** (A) The multiple modification models and sites retrieved from PhosphoSitePlus database. (B) Subcutaneous tumor volumes in experimental groups. Scale bars: 1 cm. (C) Tumor growth curves. (D) Tumor weights at endpoint. (E) IHC staining results of AKR1B10 in different groups (the scale bar represents 20 μm). Data represent mean ± SD; ***p < 0.001, ns, no significance.


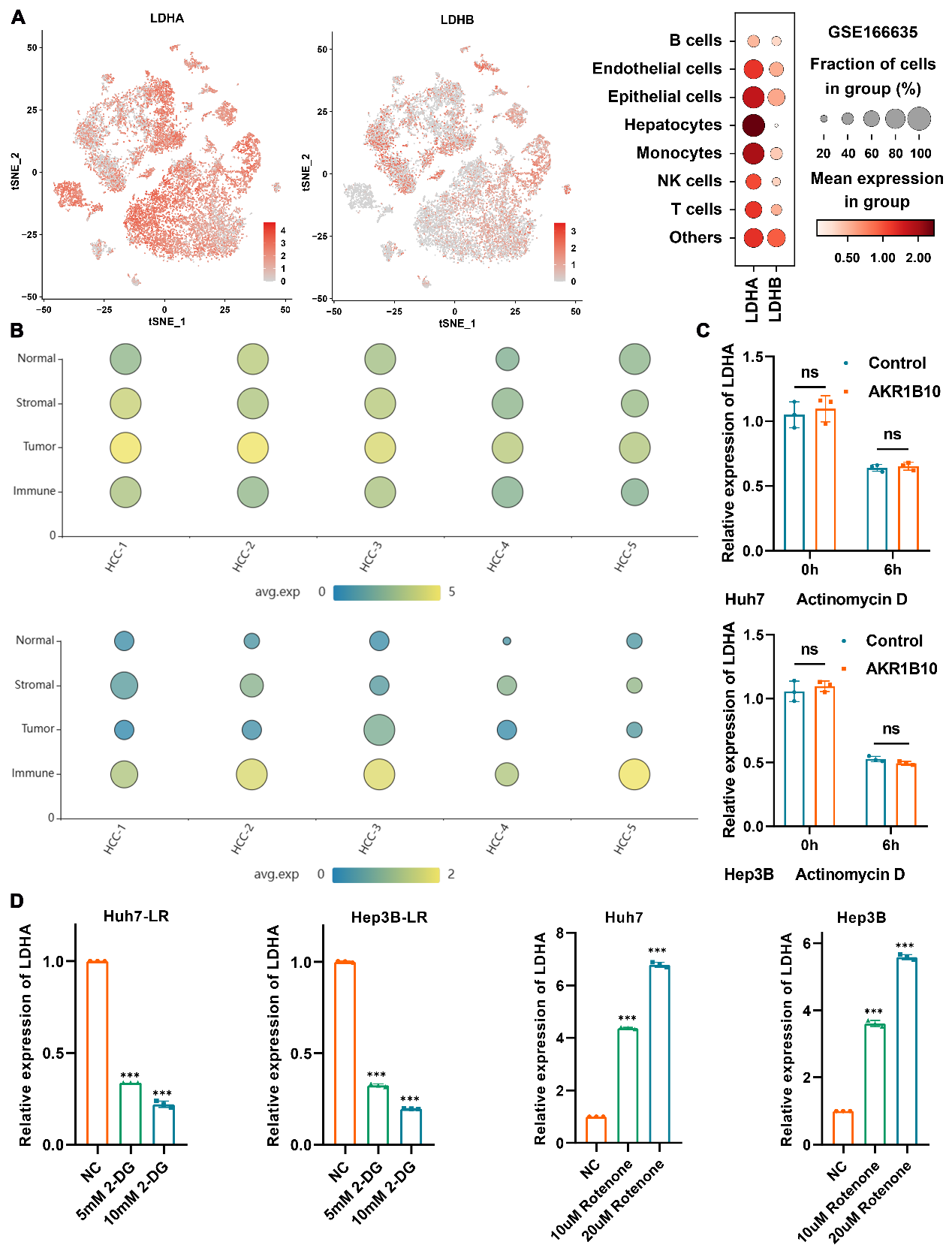


**Figure S7. Integrated analysis of LDHA with multi-omics data.** (A) The basic expression of LDHA and LDHB among different cell populations in scRNA-seq dataset GSE166635. (B) The distribution of LDHA in different IHC area in spatial transcriptomics data. (C) qRT-PCR was performed to test LDHA mRNA stability in HCC cells that stably overexpression of AKR1B10, and Actinomycin D was used to block the transcription of AKR1B10. (D) The relative mRNA expression levels of LDHA in HCC cells that stably overexpression of AKR1B10 treated with 2-DG or LR HCC cells that stably suppression of AKR1B10 treated with Rotenone. The asterisks represent the statistical p-value (*** p < 0.001, ns, no significance).

**
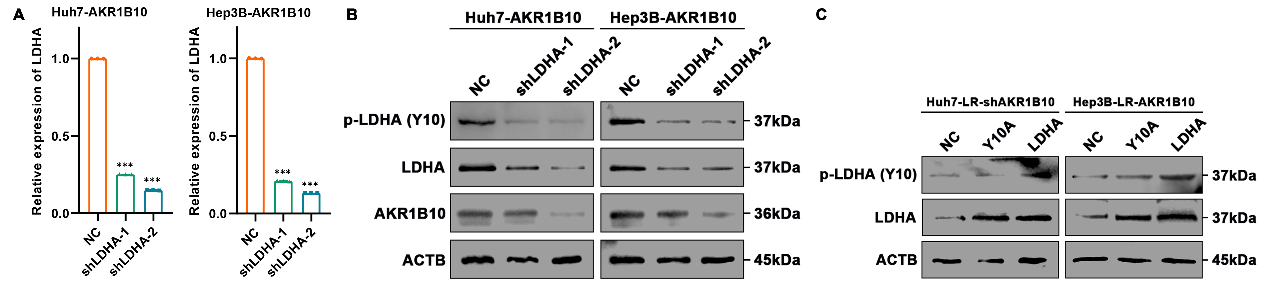
**

**Figure S8. The phosphorylation of the LDHA-Y10 is crucial for AKR1B10 mediated lenvatinib resistance in HCC.** (A) qRT-PCR to test the efficacy of shRNAs to knock down the expression of LDHA in HCC cells that stably overexpression of AKR1B10. (B) WB to test the efficacy of shRNAs to knock down the expression of LDHA, p-LDHA (Y10) and AKR1B10 in HCC cells that stably overexpression of AKR1B10. (C) WB to test the efficacy of LDHA and LDHA Y10A to reinforce the expression of LDHA and p-LDHA (Y10) in HCC cells that stably knockdown the AKR1B10. The asterisks represent the statistical p-value (*** p < 0.001).


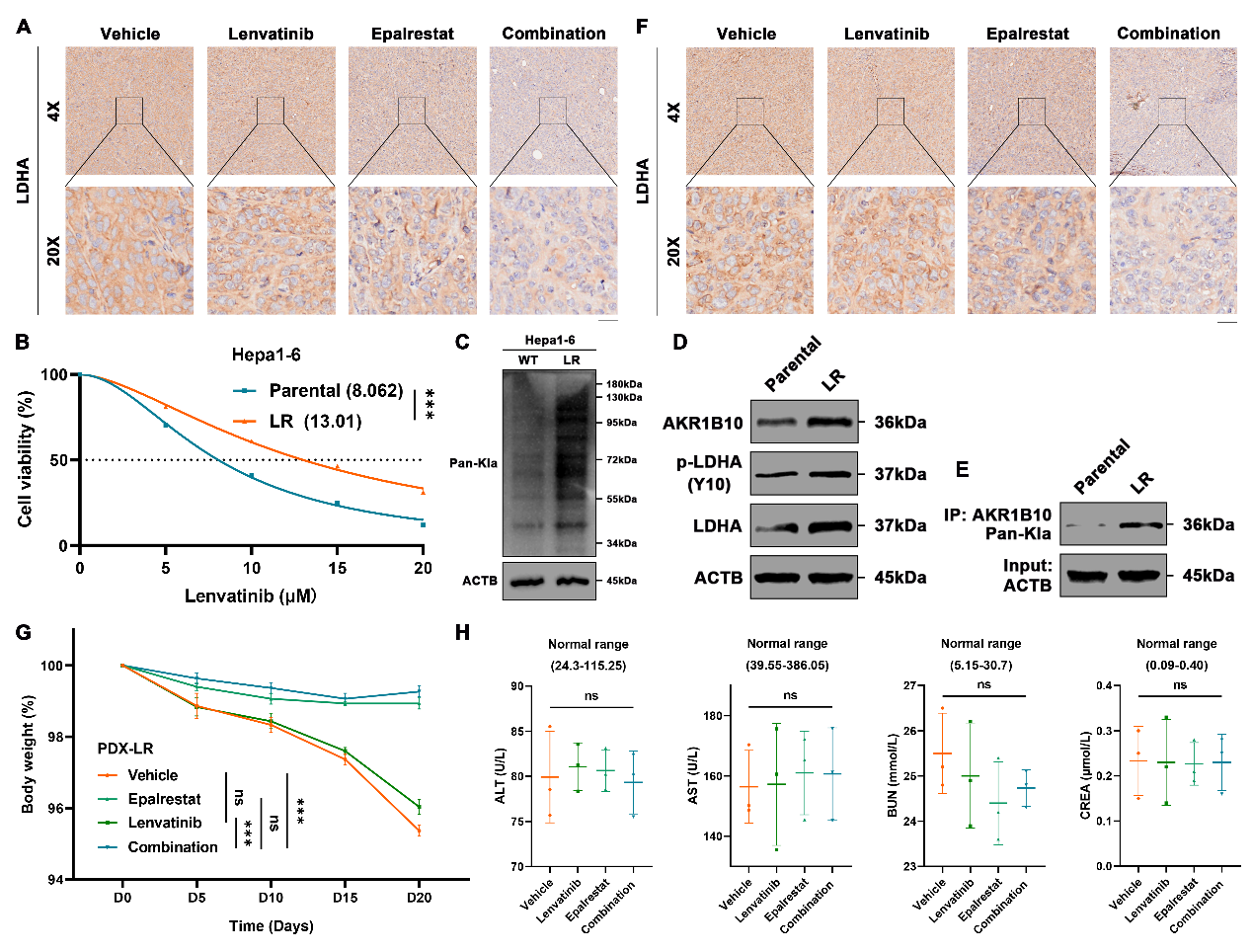


**Figure S9. IHC results of LDHA variation and constructing Hepa1-6 LR cell lines.** (A) IHC staining results of LDHA in different groups from the subcutaneous models constructed by Huh7 LR cells (the scale bar represents 20 μm). (B) IC50 values for parental and LR Hepa1-6 cell line treated with lenvatinib. (C) Western blot analysis comparing Pan-Kla (Pan-lysine lactylation) levels in parental and LR Hepa1-6 cells. (D) Protein levels of AKR1B10, LDHA and p-LDHA (Y10) between the parental and LR Hepa1-6 cells. (E) Western blot analysis of Pan-Kla levels after immunoprecipitation with anti-AKR1B10 antibody using lysates from parental and Hepa1-6 LR cells. (F) IHC staining results of LDHA in different groups from the subcutaneous models constructed by Hepa1-6 LR cells (the scale bar represents 20 μm). (G) Changes in the percentage of mice received the indicated treatments. (H) Alanine aminotransferase (ALT), aspartate aminotransferase (AST), blood urea nitrogen (BUN) and creatinine (CREA) levels in PDX mice receiving the indicated treatments. The asterisks represent the statistical p-value (*** p < 0.001, ns, no significance).


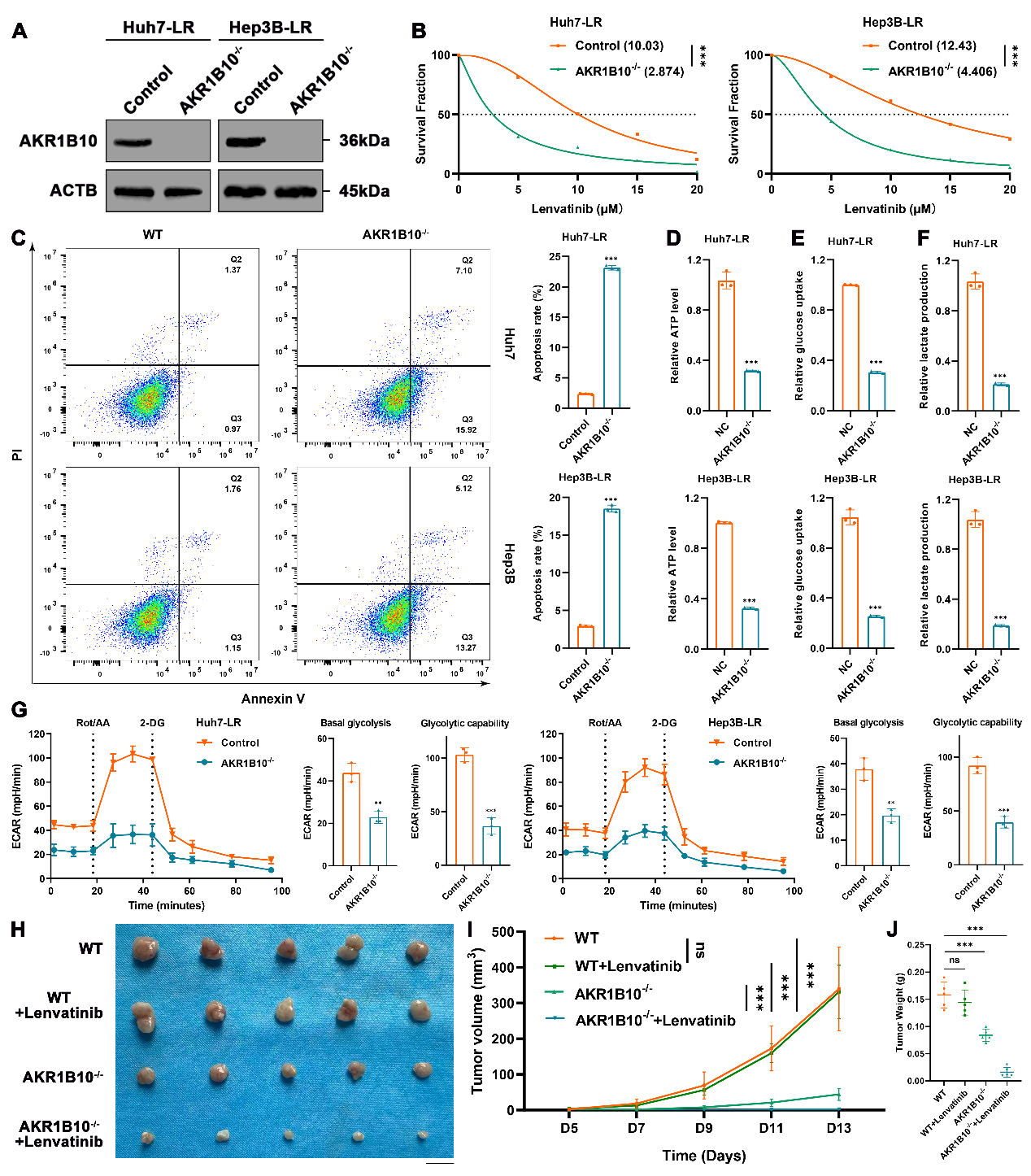


**Figure S10. Verification the role of AKR1B10 in HCC LR in vivo or in vitro using CRSIPR-CAS9 system.** (A) WB assessing AKR1B10 knockout efficiency in HCC LR cells. (B) IC50 values of lenvatinib in LR HCC cells with stable AKR1B10 knockout. (C) Apoptosis rates in AKR1B10-knockout LR HCC cells after lenvatinib treatment. (D-F) Intracellular ATP levels, intracellular glucose levels, and extracellular lactate production in AKR1B10-knockout LR HCC cells. (G) Extracellular acidification rate profiles comparing basal glycolysis and glycolytic capacity in AKR1B10-knockout LR HCC cells. (H-J) Tumor volumes/growth curves/weights in mice bearing WT or AKR1B10^-/-^ Huh7 LR xenografts received lenvatinib treatment (n=5/group). The asterisks represent the statistical p-value (** p < 0.01, *** p < 0.001, ns, no significance).
